# Triggering of high-speed neurite outgrowth using an optical microheater

**DOI:** 10.1038/srep16611

**Published:** 2015-11-16

**Authors:** Kotaro Oyama, Vadim Zeeb, Yuki Kawamura, Tomomi Arai, Mizuho Gotoh, Hideki Itoh, Takeshi Itabashi, Madoka Suzuki, Shin’ichi Ishiwata

**Affiliations:** 1 Department of Physics, School of Advanced Science and Engineering, Waseda University, 3-4-1 Okubo, Shinjuku-ku, Tokyo 169-8555, Japan; 2 Department of Cell Physiology, The Jikei University School of Medicine, 3-25-8 Nishi-shinbashi, Minato-ku, Tokyo 105-8461, Japan; 3 Institute of Theoretical and Experimental Biophysics, Russian Academy of Sciences, Pushchino, Moscow Region 142292, Russia; 4 Institute of Medical Biology, Agency for Science, Technology and Research (A*STAR), 8A Biomedical Grove, #06-06 Immunos, Singapore 138648, Singapore; 5 WASEDA Bioscience Research Institute in Singapore (WABIOS), 11 Biopolis Way, #05-02 Helios, Singapore 138667, Singapore; 6 Organization for University Research Initiatives, Waseda University, #304, Block 120-4, 513 Wasedatsurumaki-cho, Shinjuku-ku, Tokyo, 162-0041 Japan

## Abstract

Optical microheating is a powerful non-invasive method for manipulating biological functions such as gene expression, muscle contraction, and cell excitation. Here, we demonstrate its potential usage for regulating neurite outgrowth. We found that optical microheating with a water-absorbable 1,455-nm laser beam triggers directional and explosive neurite outgrowth and branching in rat hippocampal neurons. The focused laser beam under a microscope rapidly increases the local temperature from 36 °C to 41 °C (stabilized within 2 s), resulting in the elongation of neurites by more than 10 μm within 1 min. This high-speed, persistent elongation of neurites was suppressed by inhibitors of both microtubule and actin polymerization, indicating that the thermosensitive dynamics of these cytoskeletons play crucial roles in this heat-induced neurite outgrowth. Furthermore, we showed that microheating induced the regrowth of injured neurites and the interconnection of neurites. These results demonstrate the efficacy of optical microheating methods for the construction of arbitrary neural networks.

In animals, neurite outgrowth results in the formation of specific synaptic contacts with spatial accuracy. Growing neurites are attracted or retracted to different external stimuli (guidance factors). While chemical cues are the most intensively studied guidance factors^1^, physical guidance has recently been proposed. Of these, optical guidance is the representative non-invasive method for inducing attraction or retraction of growing neurites^2^,^3^,^4^,^5^,^6^,^7^,^8^,^9^,^10^. Various studies have utilized laser beams of different wavelengths to induce effective and accurate regulation of neurite outgrowth; however, the critical parameters and biological mechanism governing optical guidance remain elusive.

Here, we focus on the use of a laser beam to generate microscopic temperature increases. Localized microscopic heating (and subsequent cooling) induces gene expression^11^, muscle contraction^12^,^13^,^14^, directional bleb formation^15^, and cellular excitations such as Ca^2+^ bursts^16^,^17^ and the induction of transmembrane electrical currents^18^,^19^,^20^,^21^. Temperature increases also accelerate cytoskeletal polymerization and molecular motor activities. Thus, the use of microscopic temperature increases has the potential to provide efficient regulation of neurite outgrowth. However, conventional optical guidance methods have neglected the use of lasers for inducing microheating.

## Results

### Triggering of neurite outgrowth with an optical microheater

**Figure 1:**
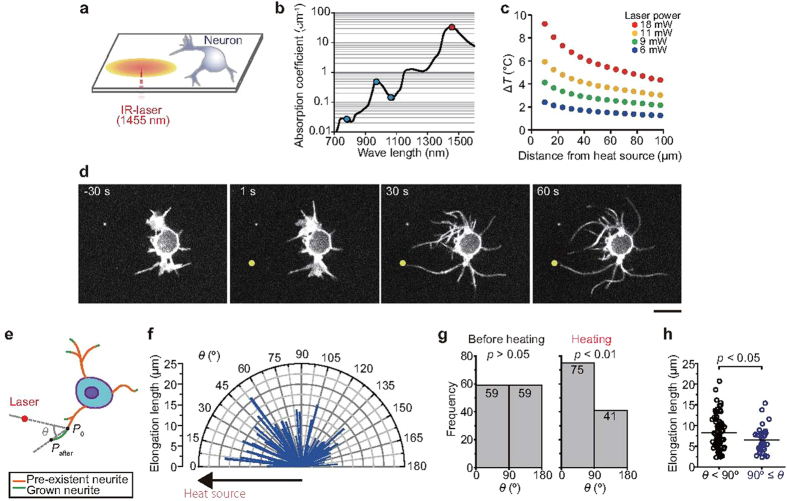
Triggering of neurite outgrowth with an optical microheater. (**a**) Schematic illustration of the experimental system. The focused infrared (IR) laser beam created a concentric temperature distribution. (**b**) Relationship between the absorption coefficient of water and the wavelength of laser light used in this study (1,455 nm, red) and in other studies of optical guidance^2^,^3^,^4^,^5^,^6^,^7^,^8^,^9^,^10^ (780–1,070 nm, blue). The absorption data were obtained from ref. 22. (**c**) Sample temperature distributions created by various laser powers. The heat source indicates the position where the IR laser beam was focused. Temperature changes were measured with a fluorescent thermosensor sheet (see Methods for details). The *T*_0_ was 36 °C. (**d**) Microheating-induced neurite outgrowth. Confocal fluorescence images of a cell treated with the membrane stain CellMask depict neurite growth toward the heat source (yellow circle). The laser power was 12 mW, the *T*_0_ was 36 °C, and heating was initiated at 0 s. Scale bar, 10 μm. See Supplementary Movie S1. (**e**) Growth angle (*θ*) was defined relative to the heat source (the position at 0° is the direction to the heat source). See Methods for detail. (**f**) Polar plots of elongation lengths during heating; n = 116 neurites. (**g**) Histogram of the number of neurites that elongated in each direction before heating (left) and during heating (right). The *p* values were determined by Chi-square test. (**h**) The length of neurite elongation in each direction. The lengths were compared using *t*-tests (*p* = 0.012). Bars indicate average values. The laser power and *T*_0_ in (**f**–**h**) were 11 mW and 36 °C, respectively.

For our experiments, we utilized a 1,455-nm infrared laser (Fig. 1a), which created a continuous heat source that was substantially more powerful than those used in previous optical guidance experiments^2^,^3^,^4^,^5^,^6^,^7^,^8^,^9^,^10^ (Fig. 1b). Indeed, the water absorption coefficient of the 1,455-nm laser light is more than 100-fold greater than that of a 1,064-nm laser^22^ (Fig. 1b). Exposure of cells to a localized temperature gradient through focusing of the 1,455-nm laser resulted in neurite outgrowth from rat hippocampal neurons [1 day *in vitro* (DIV)] (Fig. 1c,d), while in the absence of laser-mediated heating, neurites incubated at a starting temperature (*T*_0_) of 36 °C exhibited frequent changes in their positions (Supplementary Movie S1). Low levels of persistent growth were observed within the first several minutes. However, when microheating [the temperature change (Δ*T*) was ~5 °C] was applied, neurites exhibited continuous growth (Fig. 1d, Supplementary Movie S1) and branching (i.e., additional neurites appeared in the middle of grown neurites) (Supplementary Fig. S1a, Movie S2). Furthermore, we observed a dramatic change in the growth cone of cells during heating; the hand-like morphology changed to a structure comprised of long and thick branches (Supplementary Fig. S1b, Movie S3). In total, 65% (75/116) of neurites elongated towards the heat source (*θ* < 90°) (Fig. 1e–g), and the average elongation length towards the heat source was 8.3 ± 3.9 μm (means ± s.d.), which was greater than that of the neurites that elongated in the opposite direction (*θ* ≥ 90°, 6.6 ± 2.9 μm) (Fig. 1h). Additionally, neurite elongation was observed in 1–8 DIV neurons (Supplementary Fig. S2). These results demonstrate that microheating triggered rapid (within 1 min) and directional neurite outgrowth.

Notably, microheating also induced neurite regrowth after injury (Supplementary Fig. S3, Movie S4). We also observed that neurites around the heat source elongated during heat treatment, and physical connections between neurites from distinct neurons occurred within 1 min (Supplementary Fig. S4, Movie S5). After cooling, the neurites shrunk but maintained the newly formed connections. We also observed the propagation of Ca^2+^ signaling among the neurons connected via microheating (Supplementary Fig. S5), indicating that synaptic connections were indeed formed between the cells. These results suggest that microheating is a powerful method for generating networks with targeted neural connections.

### Temperature-sensitivity of the neurite outgrowth induced under a temperature gradient

**Figure 2:**
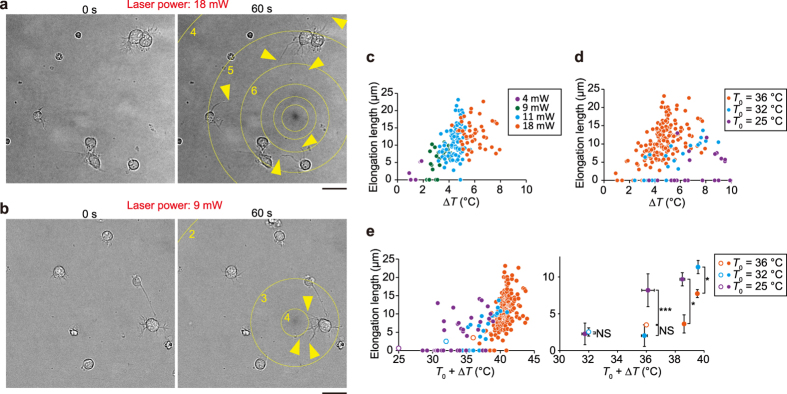
Neurite outgrowth in the presence of different temperature gradients. (**a**,**b**) Bright-field optical micrographs of neurons heated by laser power of 18 mW (**a**) or 9 mW (**b**) for 60 s. Yellow arrowheads indicate neurites that elongated during heating. Circles indicate the isotherm (displayed >10 μm from the heat source). Numbers indicate the temperature change (Δ*T*; °C) from the *T*_0_. Scale bars, 20 μm. The *T*_0_ was 36 °C. The inhomogeneous brightness of the background was subtracted (see Supplementary Methods). See Supplementary Movies S6 and S7. (**c**) The relationship between Δ*T* and the elongation length after 60 s heat treatment at a *T*_0_ of 36 °C. Each colour corresponds to a different laser power. (**d**) The relationship between Δ*T* and the elongation length after 60 s at different *T*_0_. (**e**) The relationship between *T*_0_ + Δ*T* and the elongation length after 60 s at different *T*_0_. Left: Raw data from heat-treated cells (closed circles, *T*_0_ + Δ*T*) and average values from cells in the absence of heating (open circles, *T*_0_). Error bars, standard error of the mean (s.e.m). The elongation lengths were compared using *t*-tests (*p < 0.05, ***p < 0.001; NS, not significant). Each colour corresponds to a distinct *T*_0_. See also Supplementary Table S1.

Next, we changed the Δ*T* used for inducing neurite outgrowth. Temperature changes could be mediated by adjustment of the laser power and/or the distance from the heat source to neurons (Fig. 1c). When the laser power was 18 mW, neurons in the observation area (within ~100 μm from the heat source) exhibited neurite outgrowth (Fig. 2a, Supplementary Movie S6). Meanwhile, neurite outgrowth was observed within ~20 μm of the heat source when the laser power was decreased to 9 mW (Fig. 2b, Supplementary Movie S7). Notably, weak heating generated asymmetric neurite outgrowth within individual cells (Fig. 2b, centre right), suggesting that the neurites of a given cell sense the local temperature changes independent of one another.

Microheating induces convection water flow^15^,^16^. Therefore, we investigated whether the convection flow itself induced neurite outgrowth by subjecting neurons to an artificial water flow prior to microheating (Supplementary Fig. S6, Movie S8). Although the artificial water flow induced by aspiration was stronger than the convection flow, the neurite elongation length observed under these conditions was indistinguishable from that of spontaneous neurite outgrowth (Supplementary Fig. S6, Movie S8). We thus concluded that the contribution of water flow to the neural outgrowth was negligible.

To determine whether the Δ*T* or the absolute temperature (*T*_0_ + Δ*T*) was the principal factor that triggered the observed outgrowth, we examined the extent of neurite growth during 1 min of heat treatment at different *T*_0_. At a *T*_0_ of 36 °C, the level of elongation increased concurrently with the increase in temperature (Δ*T*) (Fig. 2c). Meanwhile, when the *T*_0_ was room temperature (25 °C), the effect of Δ*T* on neurite outgrowth was less than that observed at a *T*_0_ of 36 °C (Fig. 2d). As depicted in Fig. 2e, the elongation length is not simply determined by *T*_0_ + Δ*T*. For example, the elongation length induced under microheating conditions of Δ*T* = 11 °C at *T*_0_ = 25 °C (*T*_0_ + Δ*T* = 36 °C) was 8.1 ± 4.5 μm, which was significantly higher than the average length of spontaneous neurite elongation (3.5 ± 2.7 μm) at *T*_0_ = 36 °C. Notably, when cells were subjected to 3 min of heat treatment, substantial neurite elongation was observed within 1 min after the temperature increase; however, during the subsequent two minutes, there was a marked decrease in the growth rate (Supplementary Fig. S7), indicating that the rapid increase in temperature was the key factor for triggering high-speed neurite outgrowth.

### Ca^2+^ influx also contributes to neurite elongation

**Figure 3:**
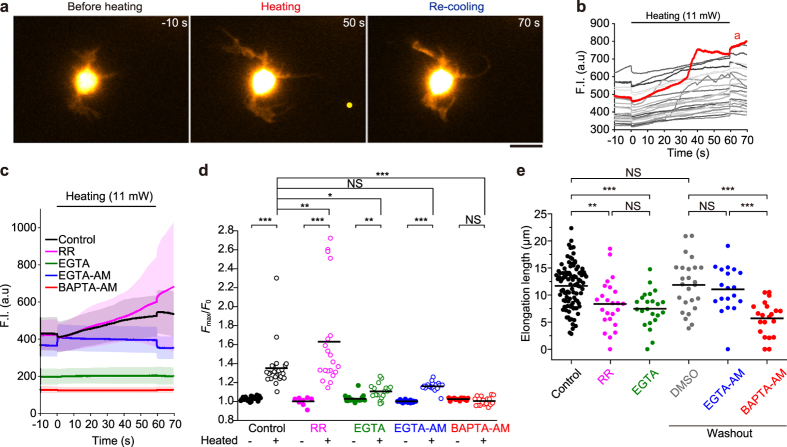
Involvement of Ca^2+^ in neurite outgrowth during microheating. (**a**) Fluorescence images of a fluo-4-loaded neuron (1 DIV). Scale bar, 10 μm. A yellow circle indicates the position of the heat source. See also Supplementary Movie S9. (**b**) Time course analysis of the fluorescence intensity (F.I.) of fluo-4. The red line indicates the value obtained from the cell in (**a**). (**c**) Time course analysis of the average F.I. in the absence of chemical agents (Control), in the presence of 30 μM ruthenium red (RR) or 1.8 mM EGTA without extracellular Ca^2+^ (EGTA), and after pre-incubation (and washout) with 30 μM EGTA-AM or 50 μM BAPTA-AM. The extracellular Ca^2+^ concentration for each group, except the EGTA group, was 1.8 mM. Error bar, s.d. Each raw data is depicted in Supplementary Fig. S8. (**d**) The maximum value of the normalized F.I. (*F*_max_/*F*_0_) during heating, or without heating (see also Supplementary Fig. S8). Bars indicate average values. The *F*_max_/*F*_0_ values of non-heated cells were compared to those of heat-treated cells using *t*-tests (**p < 0.01, ***p < 0.001; NS, not significant). Each *F*_max_/*F*_0_ value for heated cells was compared to that for the control by one-way ANOVA with Tukey-Kramer tests (*p < 0.05, **p < 0.01, ***p < 0.001; NS, not significant). (**e**) Elongation lengths of neurites after the 60 s heat treatment under each condition. Bars indicate average values. The *T*_0_ was 36 °C, and the laser power was 11 mW. The Δ*T* was 4.9 ± 0.4 °C (means ± s.d.). Elongation lengths were compared using one-way ANOVA with Tukey-Kramer tests (*p < 0.05, ***p < 0.001; NS, not significant). See also Supplementary Table S2.

Next, we investigated the effects of Ca^2+^ on neurite outgrowth during heating. It has been shown that movement of the growth cone is regulated by a Ca^2+^ influx^23^. We initially focused on thermosensitive transient receptor potential (TRP) channels, which sense temperature changes and trigger Ca^2+^ influxes^24^. The TRP-mediated Ca^2+^ influx is predicted to play a role in the optical guidance of the growth cone^25^. Fluorescence Ca^2+^ imaging detected that the concentrations of intracellular Ca^2+^ ([Ca^2+^]_i_) increased during the microheating (Fig. 3a,b, Supplementary Fig. S8, Movie S9). Contrary to the predicted TRP-mediated Ca^2+^ influx, however, the [Ca^2+^]_i_ increase was not suppressed, but enhanced, by treatment with 30 μM ruthenium red (a universal inhibitor of TRP channels^26^) (Fig. 3c,d, Supplementary Fig. S8), and this increase might have been due to the inhibition of mitochondrial Ca^2+^ uptake resulting from ruthenium red treatment^27^. Meanwhile, chelation of extracellular Ca^2+^ with 1.8 mM EGTA resulted in a significant decrease in the [Ca^2+^]_i_ prior to heating, and suppression of the [Ca^2+^]_i_ increase during heating. To further confirm the role of Ca^2+^ influxes, we examined the effects of chemical inhibitors of Ca^2+^ signalling on the levels of neurite elongation triggered by microheating. In the absence of chemical agents (control cells), neurites were observed to elongate by 11.7 ± 4.0 μm (n = 100 cells) after 1 min microheating (Fig. 3e). While neurite elongation was partly suppressed in the presence of 30 μM ruthenium red (8.4 ± 4.5 μm, n = 24) (Fig. 3e), inhibition of the Ca^2+^ influx by treatment with 1.8 mM EGTA appreciably suppressed the neurite growth (7.5 ± 3.4 μm, n = 23). To chelate intracellular Ca^2+^, we treated cells with EGTA-AM or BAPTA-AM, both of which decreased basal [Ca^2+^]_i_ (Supplementary Fig. S8) and diminished the [Ca^2+^]_i_ increase induced by high potassium stimulation (Supplementary Fig. S9). Although EGTA-AM did not affect the [Ca^2+^]_i_ increase during heating or the elongation length (11.1 ± 4.2 μm, n = 19), BAPTA-AM suppressed the [Ca^2+^]_i_ increase during heating and resulted in decreased elongation lengths (5.7 ± 3.1 μm, n = 21) (Fig. 3c–e). The distinct effects of these compounds might be due to the fact that the Ca^2+^ binding rate of BAPTA is ~40 times greater than that of EGTA^28^, and/or that BAPTA treatment results in depolymerisation of actin filaments (Supplementary Fig. S10)^29^. In summary, we conclude that the Ca^2+^ influx contributes to, but is not essential for, neurite elongation.

### Analysis of the cytoskeletal components involved in neurite elongation during microheating

**Figure 4:**
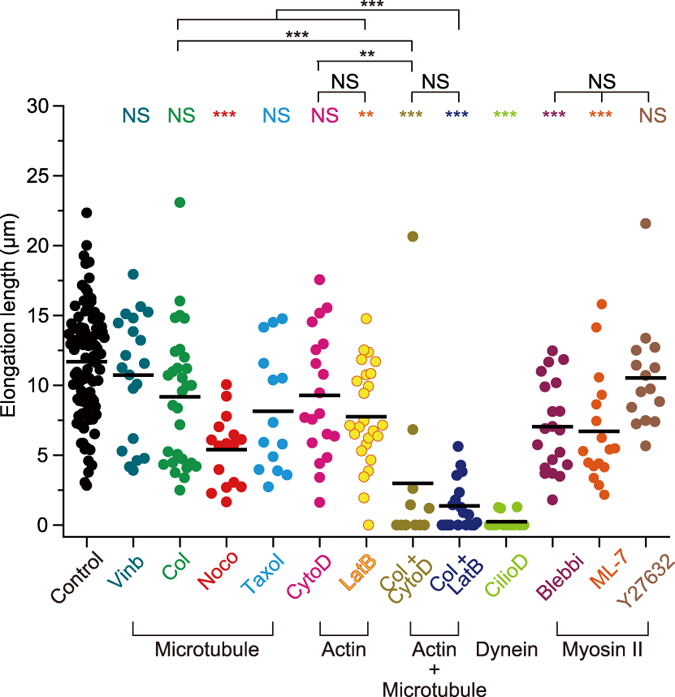
Involvement of cytoskeletal components in neurite outgrowth during microheating. Summary of the elongation lengths of neurites after 60 s heat treatment in the absence of chemical agents (Control), and in the presence of 10 nM vinblastine (Vinb), 30 μg·mL^−1^ colchicine (Col), 10 μM nocodazole (Noco), 20 μM taxol, 10 μM cytochalasin D (CytoD), 10 μM latrunculin B (LatB), 30 μg·mL^−1^ colchicine and 10 μM cytochalasin D (Col + CytoD), 30 μg·mL^−1^ colchicine and 10 μM latrunculin B (Col + LatB), 25 μM ciliobrevin D (CilioD), 25 μM blebbistatin (Blebbi), 20 μM ML-7, or 10 μM Y-27632. Bars indicate average values. The laser power was 11 mW, the Δ*T* was 4.9 ± 0.4 °C (means ± s.d.), and the *T*_0_ was 36 °C. Elongation lengths were compared by one-way ANOVA with Tukey-Kramer tests (**p < 0.01, ***p < 0.001; NS, not significant).

Directional changes of neurite outgrowth result from altered activities and distributions of actin networks and microtubules^30^. We therefore investigated the involvement of cytoskeletal components in the elongation of neurites during microheating. Treatment with a substoichiometric concentration of vinblastine (10 nM), which inhibits microtubule polymerization but does not affect the existing microtubule network^31^,^32^ (Supplementary Fig. S11), had no effect on the elongation length of neurites subjected to microheating (10.7 ± 4.5 μm, n = 20) (Fig. 4). Thus, microtubule sliding plays a substantial role in the enhanced neurite elongation. In contrast, treatment with 30 μg·mL^−1^ colchicine (depolymerizer of microtubules) and 20 μM paclitaxel (taxol; stabilizer of microtubules), respectively, resulted in only slight (but not statistically significant) decreases in neurite elongation lengths (9.2 ± 4.8 μm, n = 30 and 8.1 ± 4.4 μm, n = 14, respectively). Likewise, the length of neurite elongation was decreased in the presence of 10 μM nocodazole (depolymerizer of microtubules) (5.4 ± 2.5 μm, n = 16). Furthermore, treatment with the actin polymerization inhibitor latrunculin B (10 μM) resulted in decreases in elongation length (7.8 ± 3.6 μm, n = 25). Although treatment with 10 μM cytochalasin D (inhibitor of actin polymerization, see Supplementary Fig. S10) did not significantly affect elongation length (9.3 ± 4.6 μm, n = 19), double treatment with colchicine and either cytochalasin D or latrunculin B strongly inhibited neurite elongation (3.0 ± 6.2 μm, n = 11, or 1.4 ± 1.8 μm, n = 19, respectively). The distinct effects produced by cytochalasin D and latrunculin B are attributable to the different effects of the reagents on actin. Cytochalasin D caps the barbed ends of F-actin, and inhibits both the polymerization/depolymerization and the direct coupling between F-actins. Besides, cytochalasin D has a potential to sever F-actin^33^. Therefore, treatment of cytochalasin D fragmented F-actin but did not reduce the amount of F-actin (Supplementary Fig. S10). On the other hand, latrunculin B binds G-actin, inhibits the polymerization and promotes the depolymerization^34^. Actually, the amount of rhodamine phalloidin-labelled F-actin was significantly decreased in the presence of latrunculin B (Supplementary Fig. S10). Meanwhile, treatment with 25 μM ciliobrevin D (inhibitor of dynein), 25 μM blebbistatin (a direct inhibitor of myosin II activity), and 20 μM ML-7 [an indirect inhibitor of myosin II activity via inhibition of myosin light chain kinase (MLCK)] diminished neurite elongation (0.2 ± 0.5 μm, n = 16, 7.0 ± 3.2 μm, n = 20, and 6.7 ± 3.9 μm, n = 17, respectively). Conversely, 10 μM Y-27632 [an indirect inhibitor of myosin II activity via inhibition of rho-kinase (ROCK)] had no effect on the elongation length compared to the control cells (10.7 ± 3.6 μm, n = 15). These results support the following conclusions: 1) neurite outgrowth induced by microheating is predominantly the result of enhanced microtubule and actin dynamics, and 2) interactions between the cytoskeletal networks and molecular motors are essential for rapid neurite elongation.

**Figure 5:**
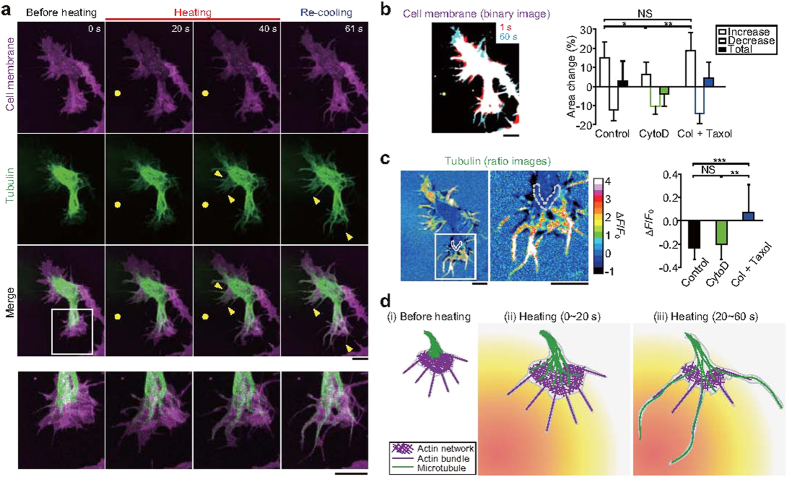
Microtubule elongation and actin-based membrane dynamics in neurites during microheating. (**a**) Confocal fluorescence images of CellMask-stained (magenta) neurons expressing tubulin-GFP (green), and merged images. Bottom panels contain magnified versions of the region highlighted in the above merged image. Yellow circles indicate the positions of the heat source. Yellow arrowheads point to areas of microtubule elongation. The *T*_0_ was 36 °C, and the laser power was 22 mW. See Supplementary Movie S10. (**b**) Left: a merged binary image of cell membranes in the absence of chemical agents [Control; same cell as in (**a**)]. Red binary images (at the first 1 s of heating) and cyan images (after 60 s of heating) are superimposed. Right: relative changes in the surface area of the growth cones in control cells, and in cells treated with 10 μM cytochalasin D (CytoD, see also Supplementary Fig. S12a) or with both 30 μg·mL^−1^ colchicine (Col) and 20 μM taxol (see also Supplementary Fig. S12b). The relative increases in surface area were compared by one-way ANOVA with Tukey-Kramer tests (*p < 0.05, **p < 0.01; NS, not significant). See also Supplementary Table S3. (**c**) Left: images depicting the ratio of GFP-tubulin in a control cell after heating to that before heating [same cell as in (**a**)], and a magnified view of the region highlighted by the white rectangle. Right: relative changes in the fluorescence intensity (Δ*F*/*F*_0_) of GFP-tubulin after heating. The Δ*F*/*F*_0_ values were obtained from the edge of the area of tubulin accumulation within the root of the growth cone before heating (the area surrounded by the grey dashed line in the figures at the left). Due to the microtubule movement from the root toward the tip, there was a decrease in Δ*F*/*F*_0_. See also Supplementary Table S4. The Δ*F*/*F*_0_ values were compared using one-way ANOVA with Tukey-Kramer tests (**p < 0.01, ***p < 0.001; NS, not significant). Scale bars in (**b**,**c**), 10 μm. (**d**) Model of neurite outgrowth during microheating. See text for the detail.

To visualize the microtubule dynamics, we utilized cells expressing tubulin-GFP. Confocal fluorescence imaging detected elongation of microtubules coupled with filopodium-like structures during microheating in 4 DIV cells (Fig. 5a,b, Supplementary Movie S10). Microtubule elongation was also observed in the presence of 10 μM cytochalasin D (Fig. 5c, Supplementary Fig. S12a, Movie S11). While microtubule dynamics were strongly inhibited by treatment with 30 μg·mL^−1^ colchicine and 20 μM taxol (Fig. 5c), elongation of certain filopodium-like structures was still observed (Fig. 5b, Supplementary Fig. S12b, Movie S12). Meanwhile, immunostaining of acetylated or tyrosinated α-tubulin demonstrated that microheating had no significant effect on tubulin modification in axons (the longest neurites) or in other minor neurites within 3 DIV cells (Supplementary Fig. S13).

To further visualize microtubule polymerization, we examined neurons expressing EB1-GFP, which binds to the plus-end of elongating microtubules. The movement velocity of EB1-GFP before heating (0.32 ± 0.09 μm·s^−1^; n = 119 comets, *T*_0_ = 36 °C) (Supplementary Fig. S14 and Movie S13) was comparable to previously reported rates of EB3-GFP movement [0.22 μm·s^−1^ in young (3–4 DIV) mouse hippocampal neurons^35^ and 0.12 μm·s^−1^ in mature (21 DIV) rat hippocampal neurons^36^, both recorded at 37 °C]. This velocity increased significantly during heating (0.49 ± 0.17 μm·s^−1^; n = 91, Δ*T* = 5.2 ± 0.6 °C), and returned to the baseline rate after re-cooling (0.30 ± 0.10 μm·s^−1^; n = 84) (Supplementary Fig. S14). These findings demonstrate that microtubule polymerization was enhanced during heating.

From these observations, we constructed a model of neurite outgrowth during microheating (Fig. 5d). Before treatment, the growth cone tips are composed of actin networks and bundles, and microtubules are located within the middle of the growth cone [(i) in Fig. 5d]^37^. During the first 20 s of heating, actin-mediated elongation of the cell membrane and tubulin polymerization are induced [(ii) in Fig. 5d]. Subsequently, the elongated microtubules induce changes in the morphology of the growth cone [(iii) in Fig. 5d], resulting in asymmetric neurite outgrowth within a single cell.

### Discussion

Our results strongly suggest that the activation of microtubule polymerization and microtubule sliding mediates neurite outgrowth during microscopic heating. Indeed, it is well documented that temperature increases initiate microtubule polymerization, and our results are consistent with studies demonstrating directional microtubule polymerization in the presence of non-microscopic, steady-state temperature gradients^38^. The molecular motor dynein generates the pushing forces within the growth cone^39^,^40^, while kinesin-1 has the potential to initiate neurite outgrowth in young (1 DIV) neurons^31^. Indeed, temperature increases enhanced the kinesin-dependent sliding velocity of microtubules^41^ and the motility of cargo transport by dynein and kinesin^42^. Dynein- and kinesin-driven neurite outgrowth could account for the microheating-induced neurite outgrowth in 1 DIV neurons in the presence of 10 nM vinblastine (Fig. 4). In contrast, neurite spreading was not observed in 4 DIV cells treated with both 30 μg·mL^−1^ colchicine and 20 μM taxol (Supplementary Fig. S12b, Movie S12), indicating that the polymerization dynamics of microtubules were predominantly responsible for neurite outgrowth induced by microheating, at least in 4 DIV neurons. Cells treated with nocodazole (10 μM) exhibited shorter neurite elongation lengths than cells exposed to 30 μg·mL^−1^ colchicine (Fig. 4), whereas treatment with each inhibitor resulted in similar levels of microtubule depolymerization at 37 °C (Supplementary Fig. S11). We suggest that colchicine might partly dissociate from tubulin during heating, which would be consistent with previous findings demonstrating that the tubulin binding activity of colchicine decreases remarkably at 45 °C^43^.

Our results also show that actin filaments and myosin II play a role in the observed neurite growth. As illustrated in Fig. 5d, and consistent with previous reports, the tip of the growth cone is composed of actin filaments^37^. Increases in temperature accelerate actin polymerization^44^, which could contribute to the elongation of neurites. Actin filament and microtubule dynamics play significant, complementary, roles in microheating-induced neurite elongation. As such, when one cytoskeletal component was chemically inhibited, the other appeared to be able to overcome this defect and rescue neurite elongation. This hypothesis is supported by the fact that, while treatment with either colchicine or cytochalasin D resulted in only weak inhibition of neurite elongation, treatment with the combination of the two compounds resulted in a strong inhibitory effect (Fig. 4). Meanwhile, myosin II is known to regulate the flow of actin filaments^45^ and microtubule bundling^46^ within growth cones. Furthermore, it is well known that the ATPase activity and the sliding velocity of actomyosin motors are activated by temperature increase^47^, which may also contribute to the temperature-sensitivity of neurite outgrowth. The distinct effects on neurite elongation observed upon treatment with ML-7 (MLCK inhibitor) and Y-27632 (ROCK inhibitor) (Fig. 4) suggest that MLCK-mediated myosin II activity is more important during neurite elongation induced by local heating than ROCK. Our results therefore indicate that cytoskeletal components and molecular motors work as thermosensors in neurons.

Neurite outgrowth was triggered by a rapid increase in temperature (Δ*T*) as opposed to the absolute temperature (*T*_0_ + Δ*T*) (Fig. 2, Supplementary Fig. S7). Therefore, the temperature-dependency of cytoskeletal polymerization alone could not account for this growth response. Microtubule polymerization is regulated by numerous microtubule plus-end tracking proteins (+TIPs)^48^. In addition, microtubule stabilizing, destabilizing, and severing proteins might also be involved in the neurite outgrowth phenotype induced by microheating. The morphological changes observed in the growth cone during heating were the result of microtubule elongation (Fig. 5a, Supplementary Movie S10), and the abnormal microtubule-dominant growth cone (i.e., elongated microtubules close to the tip of growth cone) was previously observed in the presence of laminin^49^. Similar microtubule elongation was also observed in the absence of the actin filament capping protein Capzb2^50^, which also functions to inhibit microtubule polymerization. Therefore, as discussed above, proteins involved in the growth and stabilization of actin filaments and microtubules, as well as the interactions between these proteins, might be involved in the neurite elongation induced by microheating. Furthermore, instantaneous Δ*T* can result in unbalanced equilibrium of tubulin polymerization/depolymerization. Investigation of these biomolecular dynamics, including the microtubule polymerization rate (Supplementary Fig. S14), is the next step in clarifying the cytoskeletal-based temperature-sensing system in neurons. It is well known that there is temperature variation within the brain^51^,^52^,^53^,^54^. However, it is currently unclear how these temperature gradients contribute to the development of local neural networks. Our results suggest that local temperature gradients could comprise a mechanism for guiding neurite growth (i.e., growth toward the warmer temperatures).

The observed heat-mediated [Ca^2+^]_i_ increase was partially consistent with our previous findings that Ca^2+^ is released from the endoplasmic reticulum (ER) in HeLa and WI-38 cells via IP_3_ receptors^16^,^17^. In this study, chelation of extracellular Ca^2+^ decreased the amplitude of the heat-induced [Ca^2+^]_i_ increase (Fig. 3c,d). This result suggests that Ca^2+^ influx through plasma membrane contributes to the [Ca^2+^]_i_ increase, which is different from what occurs in HeLa and WI-38 cells. However, chelation of extracellular Ca^2+^ also decreased the [Ca^2+^]_i_ prior to heating (Supplementary Fig. S8), which could have led to decreased levels of [Ca^2+^] in the ER. The dominant source of Ca^2+^, as well as the relevant Ca^2+^ channels, should be investigated in the further study. We would also like to note that the observed neurite outgrowth triggered by microheating may be a distinct phenomenon from the growth cone guidance examined by other optical methods^2^,^3^,^4^,^5^,^6^,^7^,^8^,^9^,^10^. While Ebbesen and Bruus (2012) hypothesized that Ca^2+^ influx via thermosensitive TRP channels might be involved in growth cone guidance^25^, our results only weakly support this conclusion. Further studies examining the effects of local temperature elevation during optical guidance are therefore necessary to clarify the underlying mechanism that governs this guidance.

In this study, we demonstrated that exposure to a relatively low power (~10 mW) 1,455 nm laser dramatically enhanced neurite outgrowth compared to optical guidance (8–200 mW, as summarized in ref. 25). Whereas optical forces are confined to the focal point, our temperature gradient reached tenths of micrometres of surface area. Therefore, our findings are strictly the result of heating, and not of the mixture (superposition) of microheating and optical force. In the presence of well-calibrated temperature gradients, we observed high-speed neurite outgrowth, followed by the formation of physical connections between neighbouring cells within 1 min. Our results showcase the potential for this optical heating approach as a simple, non-invasive tool for manipulating neural networks.

## Methods

### Cell culture

Hippocampal neurons were dissected from 18–19 day-old rat embryos and cultured for 12–24 h before observation. For imaging of microtubules, cells were cultured for 4 d to allow for sufficient levels of gene expression. In some experiments, 2–8 DIV neurons were used, as described in figure legends. Further details are described in Supplementary Methods. All experimental procedures conformed to the Guidelines for Proper Conduct of Animal Experiments approved by the Science Council of Japan, and were performed according to the Regulations for Animal Experimentation at Waseda University.

### Optical setup

The microscopy method using a microheating system was previously described^12^. An iXon EM+ 897 electron multiplying charge coupled device (EM-CCD) camera (Andor Technology Ltd., Belfast, UK) was mounted on an Olympus IX-70 inverted microscope (Olympus, Tokyo, Japan). The thermosensitive fluorescent dye Europium (III) thenoyltrifluoroacetonate trihydrate (Eu-TTA), the fluorescent Ca^2+^ indicator fluo-4, and Alexa Fluor 488, 555, 594, and rhodamine were excited using a SPECTRA Light Engine (377/50 nm, 485/20 nm, and 549/15 nm; Lumencor Inc., Beaverton, OR, USA) and observed with a PlanApo N 60×/1.45 oil immersion objective lens (Olympus, Tokyo, Japan). The DM505 (Olympus) and BA515IF (Olympus), and FF562-Di02 (Semrock Inc., Rochester, NY, USA) and BA580IF (Olympus) dichroic mirror and emission filter sets were used for visualization of Eu-TTA, fluo-4, and Alexa Fluor 488, and for Alexa Fluor 555, 594, and rhodamine, respectively. In dual immunostaining experiments (described below), FF02-520/28 emission filter (Semrock Inc.) was used to separate the fluorescence of Alexa Fluor 488 from that of other dyes (Alexa Fluor 555 and 594).

Confocal fluorescence images were captured using a CSU-X1 confocal unit (Yokogawa Electric Co., Tokyo, Japan) and the iXon EM+ 897 EM-CCD camera (Andor Technologies Ltd.) attached to the Olympus IX-70 microscope. The excitation wavelengths were 488 nm (Sapphire, Coherent Inc., Santa Clara, CA, USA) and 561 nm (Sapphire, Coherent Inc.). The confocal unit contained a YOKO-T405/488/561 dichroic mirror (Semrock Inc.), and YOKO-FF01-520/35 and YOKO-FF01-617/73 emission filters (Semrock Inc.). The excitation and emission light passed through a FF409-Di03 dichroic mirror (Semrock Inc.) within the microscope.

The local temperature around candidate cells was increased by irradiation using a focused infrared laser beam (λ = 1,455 nm; KPS-STD-BT-RFL-1455-02-CO; Keopsys, Lannion, France), and the duration of irradiation was controlled by a mechanical shutter (SSH-C4B, Sigma Koki, Tokyo, Japan). The intensity of the laser at the level of the sample was measured after passage through the objective lens with an LM-3 thermal disk sensor and a FieldMaster power meter (Coherent Inc.).

### Local temperature measurements

Changes in local temperature were calculated by measuring the thermal quenching of the fluorescence intensity of the dye Eu-TTA (Acros Organics, Pittsburgh, PA, USA). For these experiments, 5 mg·mL^−1^ Eu-TTA and 10 mg·mL^−1^ poly(methyl methacrylate) (PMMA; MW = 15,000; Sigma-Aldrich Co., St. Louis, MO, USA) dissolved in acetone were spin-coated on glass dishes^14^,^17^. The temperature sensitivity of the fluorescence intensity was −4.1% °C^−1^ at 36 °C.

### Live cell imaging

With the exception of fluorescence imaging experiments and experiments using chemical agents, neurons were observed in the same medium used for culturing (Supplementary Methods). The solution temperature was adjusted during microscopy using a thermostatically controlled incubator mounted on the sample stage (INUG2-ONICS; Tokai Hit Co., Shizuoka, Japan). To stabilize the culture temperature, neurons were incubated on the microscope for 10 min prior to observations, and observations were performed within 1 h. The details are described in the Supplementary Methods.

### Immunostaining

F-actin was stained with rhodamine-phalloidin (Life Technologies, Thermo Fisher Scientific Inc., Waltham, MA, USA). Acetylated, tyrosinated, and total α-tubulin were stained with anti-acetylated α-tubulin antibody (ab24610, Abcam plc, Cambridge, UK), anti-tyrosinated α-tubulin antibody (YL1/2) (ab6160, Abcam plc), and anti-α-tubulin antibody (ab15246, Abcam plc), respectively. The details are described in the Supplementary Methods.

### Neurite analyses

Neurite tip positions were tracked manually with ImageJ software (National Institutes of Health, Bethesda, MD, USA). For analysis of directional growth (in Fig. 1e–g), growing neurites (>~2 μm), except for non-attached fluctuating neuritis, were measured. The directional angle was defined relative to the heat source (the position at 0° was nearest to the heat source) in the following order: the neurite edge points before and after heating (60 s) were defined as “*P*_0_” and “*P*_after_”, respectively (see in Fig. 1e). The directional angle of a grown neurite was defined as the angle between a line from “*P*_0_” to “*P*_after_” and another line from the laser heat source to “*P*_0_”. To determine the angle of spontaneous neurite outgrowth before heating (Fig. 1g), the neurite edge points at 60 s and 0 s before heating were defined as “*P*_0_” and “*P*_after_”, respectively. In other analyses, the neurite exhibiting the largest amount of growth on each cell was utilized as a representative neurite.

### Ca^2+^ analyses

For analysis of [Ca^2+^]_i_ dynamics, the fluorescence intensity of fluo-4 was measured using ImageJ software. Areas where moving neurites were absent were used as regions of interest (ROI) to reduce the noise. The basal fluorescence intensity of fluo-4 (*F*_0_) was defined as the average intensity for the first 1 s of observation. *F*_max_ was defined as the maximum fluorescence intensity during 2–60 s of heating. For experiments using KCl treatments (Supplementary Fig. S9), *F*_0_ was defined as the average intensity at 1 s prior to application of KCl, and *F*_max_ was defined as the maximum fluorescence intensity during and after treatment with KCl.

### Analyses of cell surface area and microtubule density

Changes in cell surface area (Fig. 5b) were determined by analysis of binary images of cells treated with CellMask stain. For these analyses, ImageJ software was used to smooth confocal fluorescence images and to reduce the noise (using the default function ‘smooth’), and images were then binarized. The threshold of binarization was determined by direct observation. Changes in microtubule density were measured by assaying for alterations in the fluorescence intensity of GFP-tubulin within ROI using ImageJ software (Fig. 5c). The ROI, a linear region 1.1 μm in width, was set to the edge of area of tubulin accumulation in the growth cone prior to heating. Δ*F* was considered the difference between the averaged fluorescence intensity at 2–4s after termination of heating and the average intensity at 0–2 s before heating, *F*_0_. In these analyses, we compensated for the effects of photobleaching.

### Immunostaining analyses

To examine the relative amounts of microtubules and F-actin in neurons, the fluorescence intensity of Alexa Fluor 488-labelled α-tubulin and rhodamine-labelled F-actin in the cell was measured using ImageJ software. To determine the ratio of acetylated or tyrosinated α-tubulin to total α-tubulin, the fluorescence intensities of Alexa Fluor 488, 555, and 594-labelled α-tubulin within neurites were measured using ImageJ. For heat-treated cells, the neurite nearest to the heat source was measured with 10 μm line-shaped ROI. For non-heat-treated cells, all neurites were measured within linear ROIs. In both cases, the width of the ROI was 1.3 μm.

### Velocity analyses

To determine the velocity of water flow or microtubule elongation, fluorescent beads or EB1-GFP comets were manually tracked using ImageJ software, respectively. The time intervals used for the analysis of the beads or comets were 100 ms or 1030 ms, respectively.

### Statistical analyses

The number of neurites elongated in each direction were compared by Chi-square test (Fig. 1g). Multi groups were compared using one-way analysis of variance (ANOVA) with Tukey-Kramer tests with OriginPro2015 software (OriginLab Co., Northampton, MA, USA). For other experiments, variance was examined using *F*-tests. When the *p* value of the variance was ≥0.05, Student’s *t*-test was used; however, when *p* < 0.05, Welch’s *t*-test was used. Chi-square test, *t*-tests and *F*-tests were performed using Microsoft Excel software.

## Additional Information

**How to cite this article**: Oyama, K. *et al*. Triggering of high-speed neurite outgrowth using an optical microheater. *Sci. Rep*. **5**, 16611; doi: 10.1038/srep16611 (2015).

## Supplementary Material

Supplementary Information

Supplementary Movie S1

Supplementary Movie S2

Supplementary Movie S3

Supplementary Movie S4

Supplementary Movie S5

Supplementary Movie S6

Supplementary Movie S7

Supplementary Movie S8

Supplementary Movie S9

Supplementary Movie S10

Supplementary Movie S11

Supplementary Movie S12

Supplementary Movie S13
