# Supplementary material for: Triggering of high-speed neurite outgrowth using an optical microheater
Source: Sci Rep. 2015 Nov 16;5:16611. doi: 10.1038/srep16611 (PMC4645119; doi:10.1038/srep16611)
Supplement: Supplementary Information [file srep16611-s1.pdf]

Supplementary Information

Triggering of high-speed neurite outgrowth using an optical microheater

Authors:

Kotaro Oyama[1,2,*], Vadim Zeeb[1,3], Yuki Kawamura[1], Tomomi Arai[1,2], Mizuho Gotoh[1], Hideki Itoh[1,4], Takeshi Itabashi[1], Madoka Suzuki[5,6,*], Shin'ichi Ishiwata[1,5,6,*]

[1]Department of Physics, School of Advanced Science and Engineering, Waseda University, 3-4-1 Okubo, Shinjuku-ku, Tokyo 169-8555, Japan.
[2]Department of Cell Physiology, The Jikei University School of Medicine, 3-25-8 Nishi-shinbashi, Minato-ku, Tokyo 105-8461, Japan
[3]Institute of Theoretical and Experimental Biophysics, Russian Academy of Sciences, Pushchino, Moscow Region 142292, Russia.
[4]Institute of Medical Biology, Agency for Science, Technology and Research (A*STAR), 8A Biomedical Grove, #06-06 Immunos, Singapore 138648, Singapore.
[5]WASEDA Bioscience Research Institute in Singapore (WABIOS), 11 Biopolis Way, #05-02 Helios, Singapore 138667, Singapore.
[6]Organization for University Research Initiatives, Waseda University, #304, Block 120-4, 513 Wasedatsurumaki-cho, Shinjuku-ku, Tokyo, 162-0041 Japan.

K.O. and V.Z. contributed equally to this work.

***Corresponding Authors**

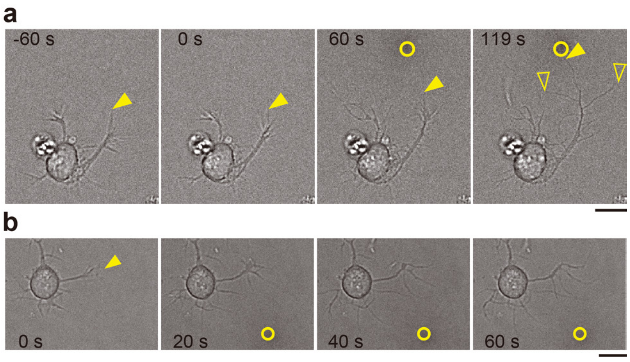

Supplementary Figure S1. Microheating induces branching of neurites. (a) Microheating induces neurite outgrowth and branching. The highlighted neurite grew in the direction of the heat source (circle). Closed arrowheads indicate the tip of the neurite and open arrowheads indicate neurite branching. See Supplementary Movie S2. (b) Morphological changes in the growth cone induced by microheating. The yellow arrowhead indicates the morphology prior to heating. During heating, the growth cone exhibited a dramatic morphological change to a structure with long, thick branches. Open circles indicate the position of the heat source. See Supplementary Movie S3. The T_0 was 36 °C, the laser power was 11 mW. Scale bars, 10 µm. Inhomogeneous brightness present in the background was subtracted (see Supplementary Methods).

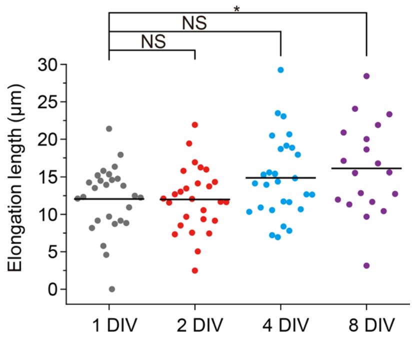

Supplementary Figure S2. Neurite outgrowth at different days *in vitro* (DIV). Summary of the elongation lengths of neurites (including filopodia-like structures, as shown in Fig. 5a) after 60 s of heat treatment. Bars indicate average values. Elongation lengths were compared to 1 DIV by one-way ANOVA with Tukey-Kramer tests (*$p < 0.05$; NS, not significant). The T_0 was 36 °C, the laser power was 11 mW. See also Supplementary Table S5.

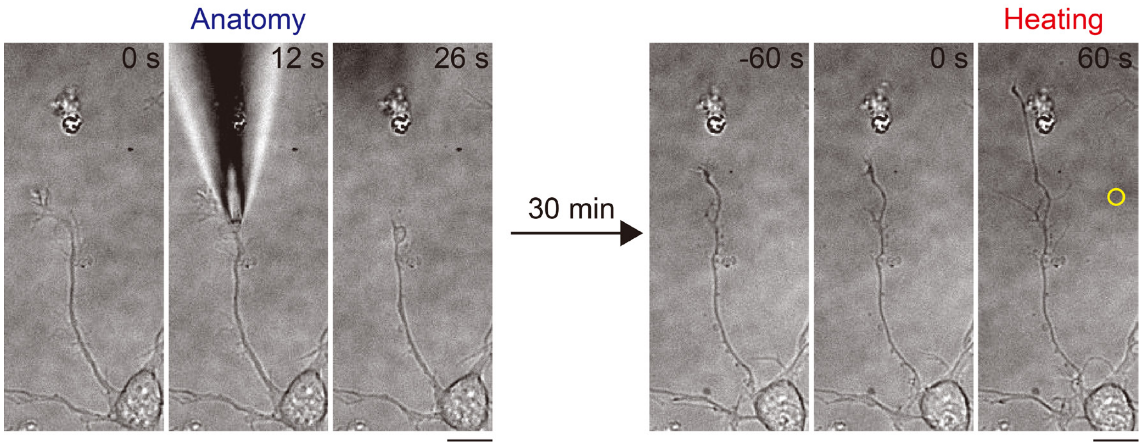

Supplementary Figure S3. Neurite regrowth triggered by microheating after injury. Left (Anatomy): images depicting the artificial injury of a neural cell. A glass capillary tube was utilized to remove a part of a neurite. Right: the injured neurite formed a small growth cone and resumed growth within 30 min after injury. Microheating for 60 s induced remarkable regrowth of the neurite. An open circle indicates the position of the heat source. The T_0 was 36 °C, the laser power was 11 mW. Scale bars, 10 µm. See also Supplementary Movie S4.

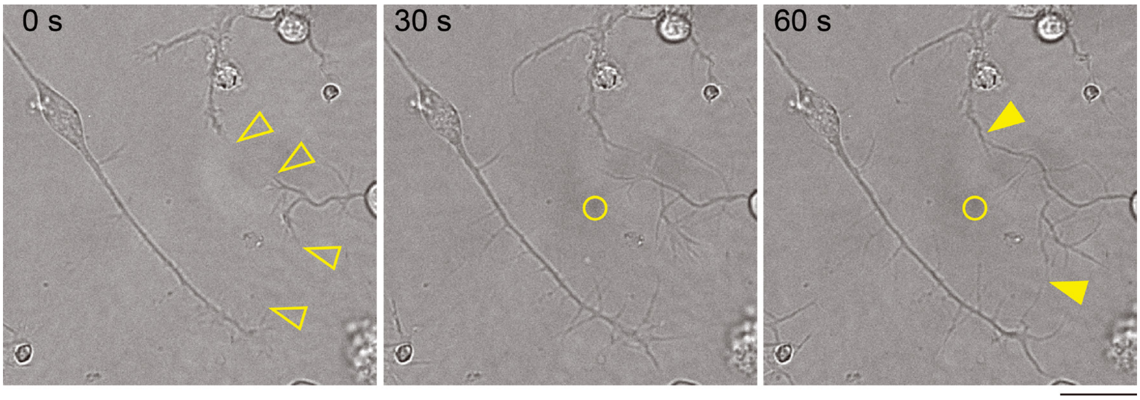

Supplementary Figure S4. Formation of physical connections in the presence of microheating. Bright-field optical micrographs showing three neurons at the top left, top right, and centre right. The neurites were not connected prior to heating (open arrowheads). Microheating induced both neurite growth and the connection of neurites from different neurons (closed arrowheads). Open circles indicate the positions of the heat source. The T_0 was 36 °C, the laser power was 11 mW. Scale bar, 20 μm. Inhomogeneous brightness present in the background was subtracted (see Supplementary Methods). See also Supplementary Movie S5.

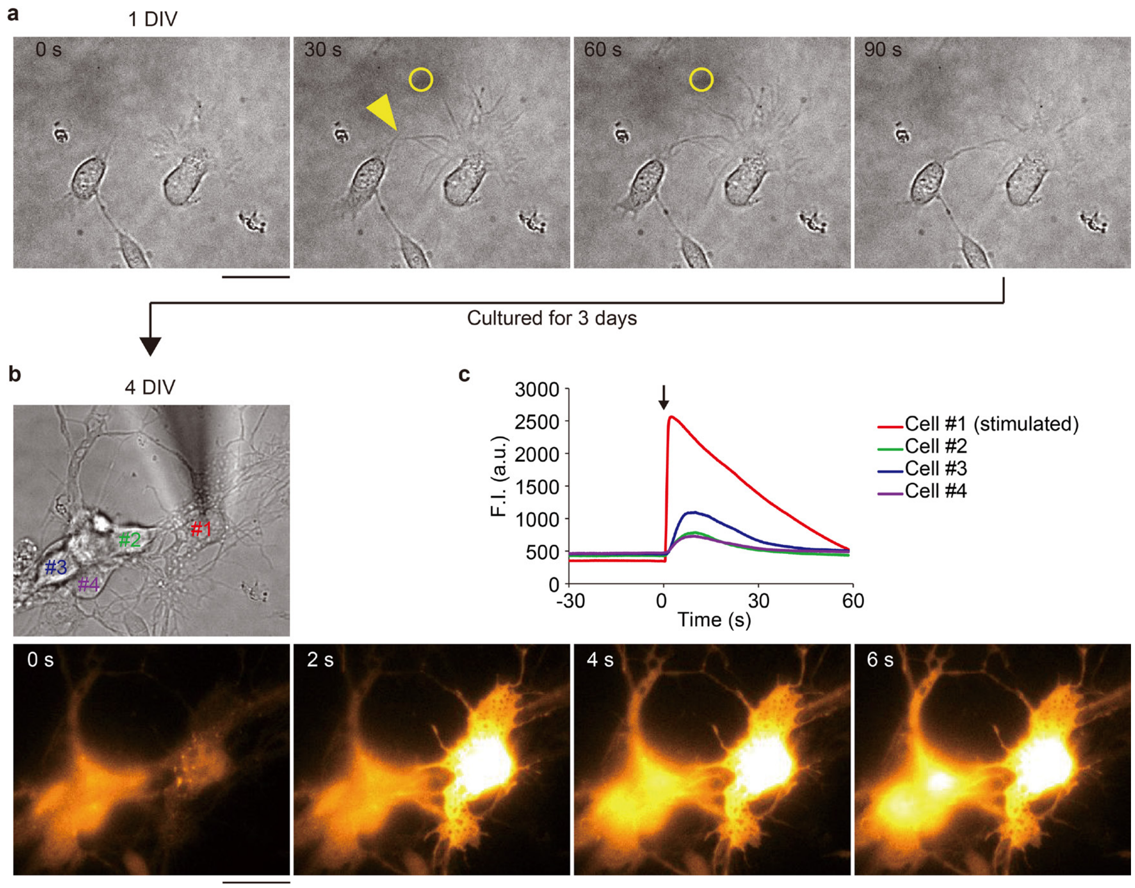

Supplementary Figure S5. Formation of synaptic connections after microheating. (a) Bright-field optical micrographs showing three neurons (1 DIV) at the left, right, and bottom. The left and right neurons were not connected prior to heating. Microheating induced the growth of a neurite from the cell on the right, and the subsequent connection of the two neurons (yellow arrowhead). Open circles indicate the positions of the heat source. The T_0 was 36 °C, the laser power was 11 mW. (b) Top: bright-field optical micrograph at the same area of (a) after culturing for 3 days. For mechanical stimulation, a glass capillary tube was placed on the cell on the right (termed "Cell #1"). Bottom: fluorescence images of fluo-4-loaded neurons. Mechanical stimulation was applied to Cell #1 at time 0 s. Increases in Ca^{2+} concentrations were then observed in the stimulated cell and in other cells (Cells #2–4). Scale bars, 20 μm. (c) Time course analysis of the fluorescence intensity (F.I.) of fluo-4 in the four neurons in (b). The arrow indicates the time at which mechanical stimulation was applied to Cell #1.

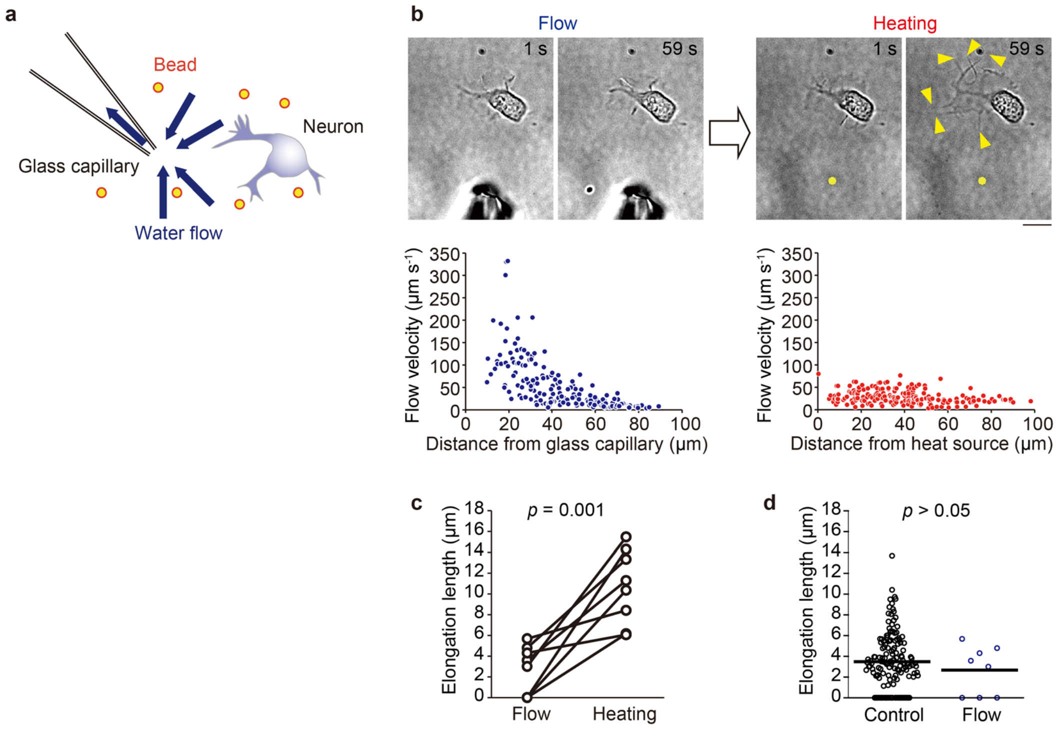

Supplementary Figure S6. Neurite outgrowth during microheating was not due to convection flow. (a) Schematic illustration of the experimental system used to create water flow around a cell. Convection-like artificial water flow was created at the tip of a glass capillary tube by negative pressure. The water flow was visualized using 1-μm (diameter) fluorescent beads. (b) Top: first, the medium was aspirated using a glass capillary tube, and water flow was induced in the absence of heating (0–60 s). Under these conditions, neurite outgrowth was not enhanced (panels 1 and 2). Conversely, microheating (0–60 s) applied near the same cell enhanced neurite outgrowth (indicated by yellow arrowheads; panels 3 and 4). The yellow circle indicates the position of the heat source. Scale bar, 10 μm. See Supplementary Movie S7. Bottom: the velocity of water flow induced by aspiration (left) or microheating (right). (c) The length of neurite elongation was 2.7 ± 2.3 μm during 60 s in the presence of water flow (n = 8), and was 10.7 ± 3.6 μm after the subsequent 60 s heat treatment (n = 8). The distance of the cells from the glass capillary tube and the heat source were approximately 20–40 μm. Although the artificial water flow was stronger than the convection flow due to microheating, the elongation length was significantly shorter under artificial conditions (p = 0.001; paired t-test). (d) The length of neurite elongation was 3.5 ± 2.7 μm in the absense of water flow after 60 s (Control, n = 151), which was not significantly different from that in the presence of water flow (p = 0.23; two-tailed t-test). Bars indicate average values. The T_0 was 36 °C, the laser power was 11 mW. See also Supplementary Movie S8.

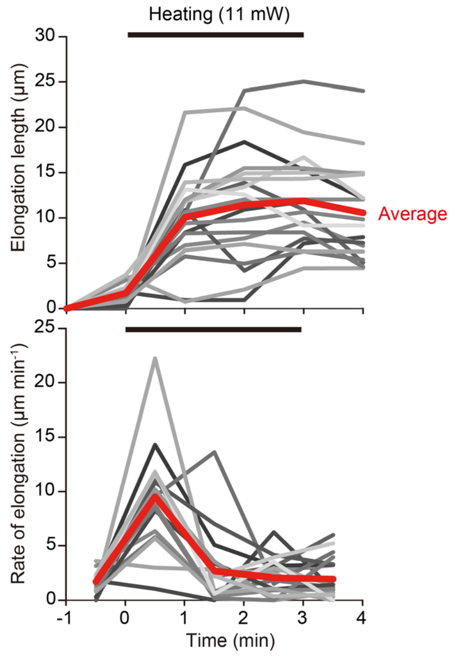

Supplementary Figure S7. Active elongation of neurites occurs just after the increase in temperature. Neurite elongation induced by microheating (11 mW) over 3 min. The highest rate of elongation was observed just after the start of heating. Gray or red lines show the individual (n = 4 cells, 19 neurites) or averaged data, respectively. The T_0 was 36 °C.

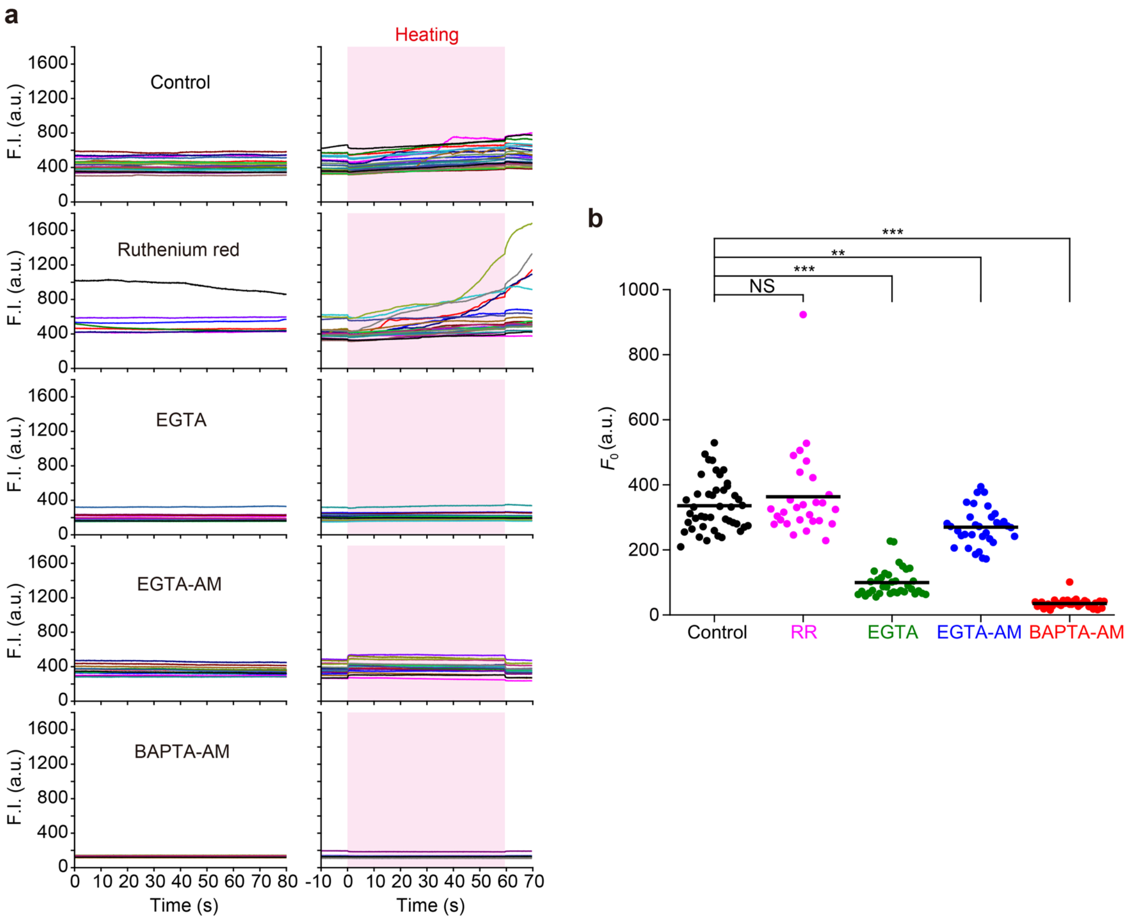

Supplementary Figure S8. Intracellular Ca^{2+} dynamics during microheating. (a) Time course analysis of the fluorescence intensity (F.I.) of fluo-4 in 1 DIV neurons in the absence of chemical agents (Control), in the presence of 30 μM ruthenium red (RR) or 1.8 mM EGTA without extracellular Ca^{2+} (EGTA), and after pre-incubation (and washout) with 30 μM EGTA-AM or 50 μM BAPTA-AM. The extracellular Ca^{2+} concentration for each group, except the EGTA group, was 1.8 mM. Left: without microheating. Right: with microheating. Red regions indicate the periods during microheating. The T_0 was 36 °C, the laser power was 11 mW. (b) Baseline F.I. (F_0) under each condition. Bars indicate average values. The values were compared to control cells by one-way ANOVA with Tukey-Kramer tests (**$p < 0.01$, ***$p < 0.001$; NS, not significant). See also Supplementary Table S2.

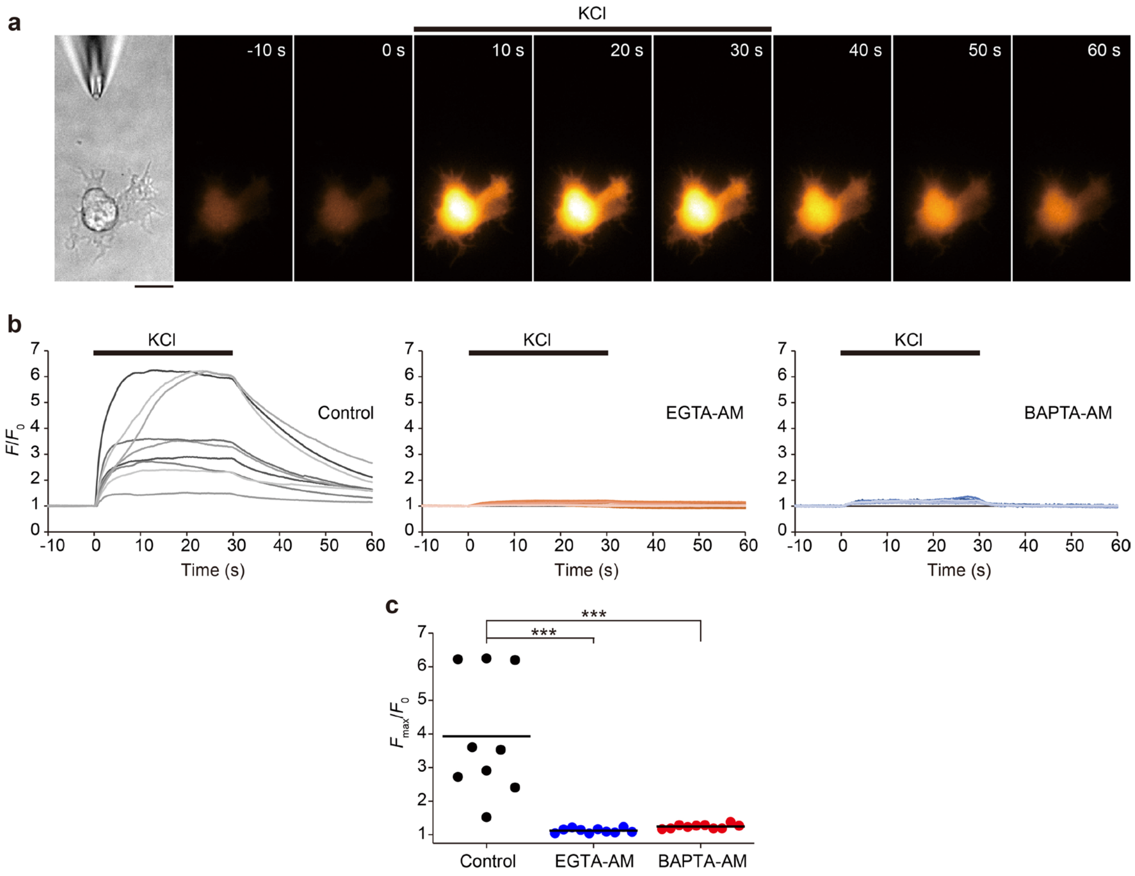

Supplementary Figure S9. Ca^{2+} increase in neurons treated with KCl. (a) Bright-field optical micrograph (left) and fluorescence images of a fluo-4-loaded neuron (1 DIV). 1 M KCl was applied to the cell using a glass capillary tube. Scale bar, 10 μm. (b) Time course analysis of the normalized fluorescence intensities (F/F_0) of fluo-4 in control (untreated) and BAPTA- (BAPTA-AM) or EGTA-loaded (EGTA-AM) cells during treatment with KCl. Bars indicate the period at which KCl was applied. (c) Maximum fluorescence intensity of fluo-4 (F_{max}/F_0) during KCl treatment. Bars indicate average values. The maximum fluorescence intensities were compared to those of untreated cells using one-way ANOVA with Tukey-Kramer tests (***$p < 0.001$; NS, not significant). See also Supplementary Table S2.

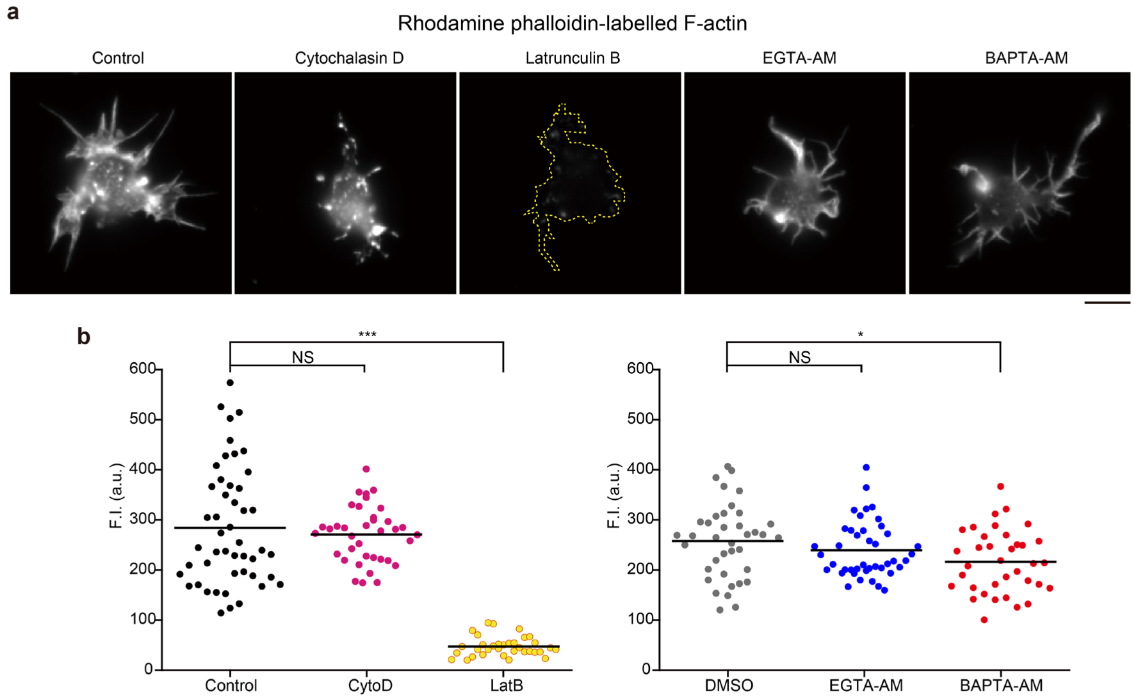

Supplementary Figure S10. Effects of chemical agents on actin filaments (F-actin) in neurons. (a) Fluorescence images of rhodamine phalloidin-labelled F-actin in neurons (1 DIV) in the absence of chemical agents (Control), in the presence of 10 μM cytochalasin D (CytoD) or 10 μM latrunculin B (LatB), and after pre-incubation (and washout) with 30 μM EGTA-AM or 50 μM BAPTA-AM at 37 °C. The yellow dashed line denotes the outline of the cell, as determined using the bright-field image. Scale bar, 10 μm. (b) Fluorescence intensity (F.I.) of rhodamine phalloidin in neurons under each condition, and after pre-incubation (and washout) with 0.1% DMSO. Bars indicate average values. The values were compared using one-way ANOVA with Tukey-Kramer tests (*$p < 0.05$, ***$p < 0.001$; NS, not significant). Although the intensity in the presence of 10 μM cytochalasin D was not different from that of the control, there was fragmentation of F-actin, as shown in (a). See also Supplementary Table S6.

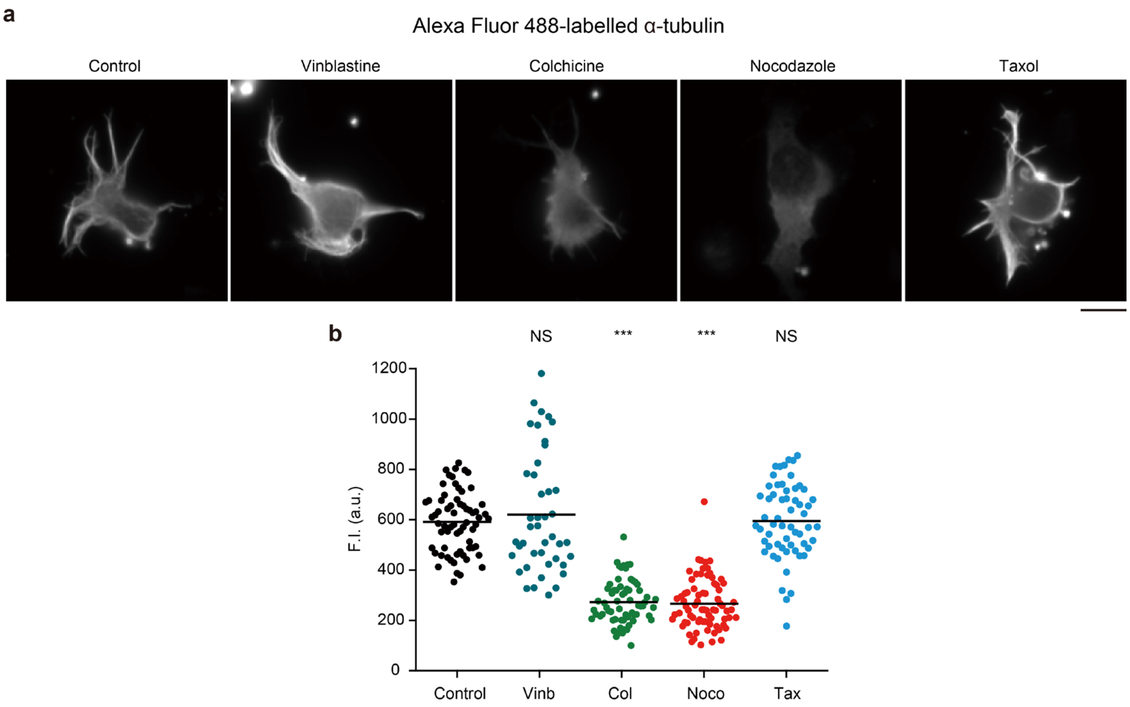

Supplementary Figure S11. Effects of chemical agents on microtubules in neurons. (a) Fluorescence images of Alexa Fluor 488-labelled tubulin in neurons (1 DIV) in the absence of chemical agents (Control), and in the presence of 10 nM vinblastine (Vinb), 30 μg·mL^{-1} colchicine (Col), 10 μM nocodazole (Noco), or 20 μM taxol (Tax) at 37 °C. Scale bar, 10 μm. (b) Fluorescence intensity (F.I.) of Alexa Fluor 488 in neurons under each condition. Bars indicate average values. The values were compared to those in control cells by one-way ANOVA with Tukey-Kramer tests (***p < 0.001; NS, not significant). See also Supplementary Table S7.

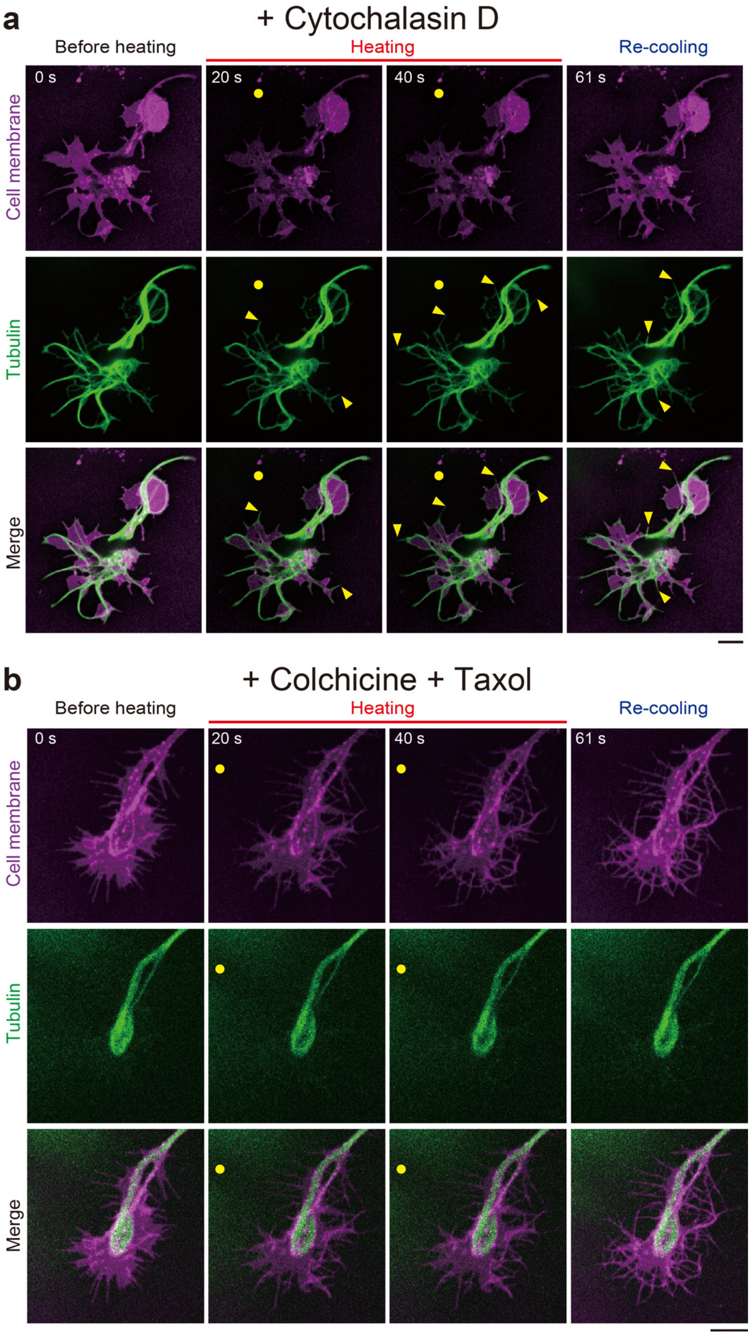

Supplementary Figure S12. Microtubule dynamics in growth cones induced by microheating. (a,b) Confocal fluorescence images of cells treated with the plasma membrane stain CellMask Orange (magenta) and expressing tubulin-GFP (green) in the presence of (a) 10 μM cytochalasin D or (b) both 30 μg·mL^{-1} colchicine and 20 μM taxol. Yellow circles indicate the position of the heat source, and yellow arrowheads indicate areas of microtubule elongation. The T_0 was 36 °C, the laser power was 22 mW. Scale bars, 10 μm. See also Supplementary Movies S11 and S12.

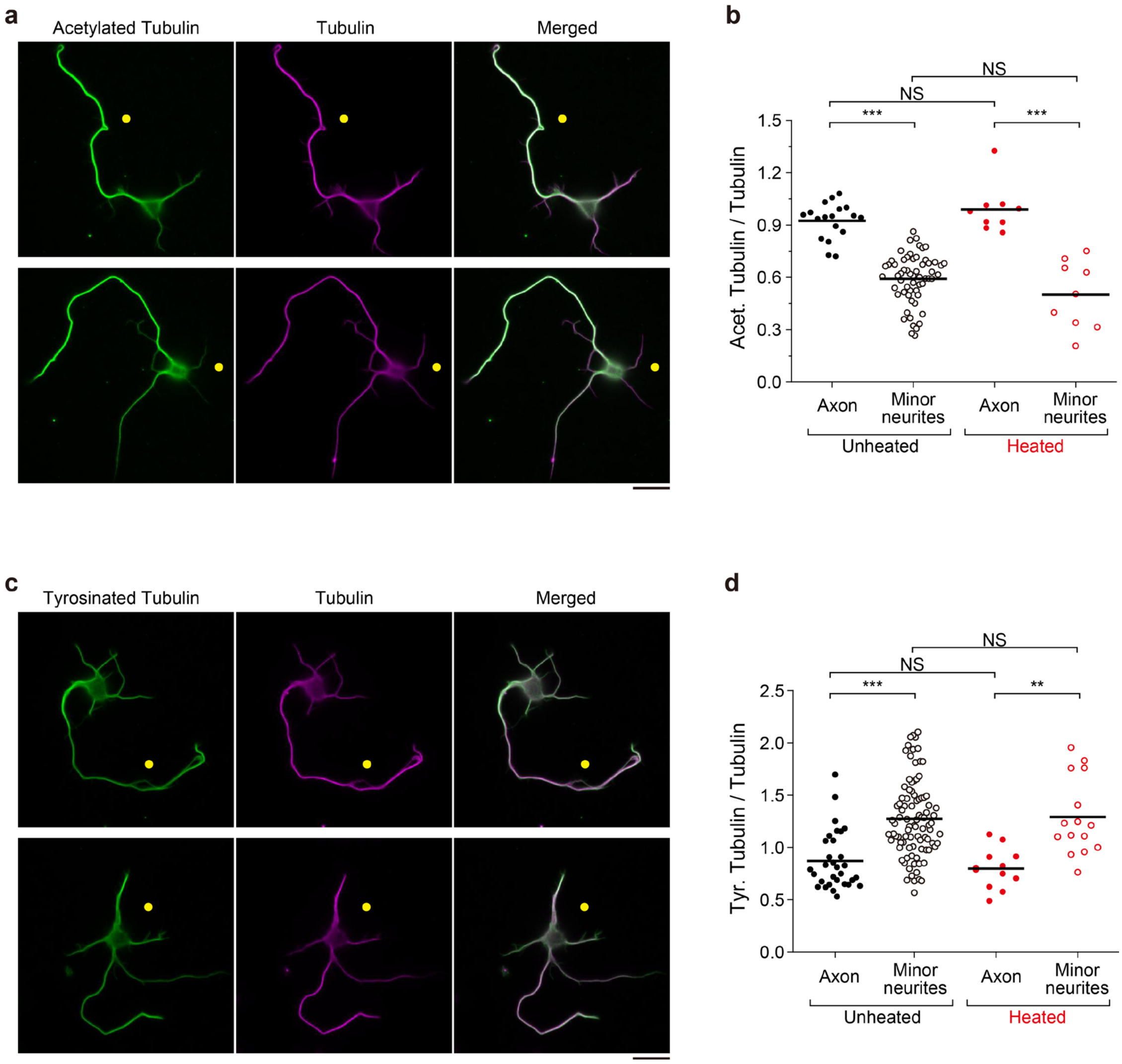

Supplementary Figure S13. Microheating does not alter the degree of post-translational modification of α-tubulins. (a) Fluorescence images of Alexa Fluor 488-labelled acetylated α-tubulin (green) and Alexa Fluor 594-labelled α-tubulin (magenta) in 3 DIV neurons. Panels on the right depict merged images. (b) Summary of the fluorescence intensity ratio of acetylated α-tubulin to total α-tubulin in axons (the longest neurite) and other minor neurites. Bars indicate average values. (c) Fluorescence images of Alexa Fluor 555-labelled tyrosinated α-tubulin (green) and Alexa Fluor 488-labelled α-tubulin (magenta) in 3 DIV neurons, and merged images. (d) Summary of the fluorescence intensity ratio of tyrosinated α-tubulin to total α–tubulin in axons and other minor neurites. Bars indicate average values. Yellow circles in (a) and (c) indicate the positions of the heat source. Scale bars, 20 μm. The T_0 was 36 °C, the laser power was 11 mW. The ratios were compared by one-way ANOVA with Tukey-Kramer tests (***p < 0.01, ***p < 0.001; NS, not significant). See also Supplementary Table S8.

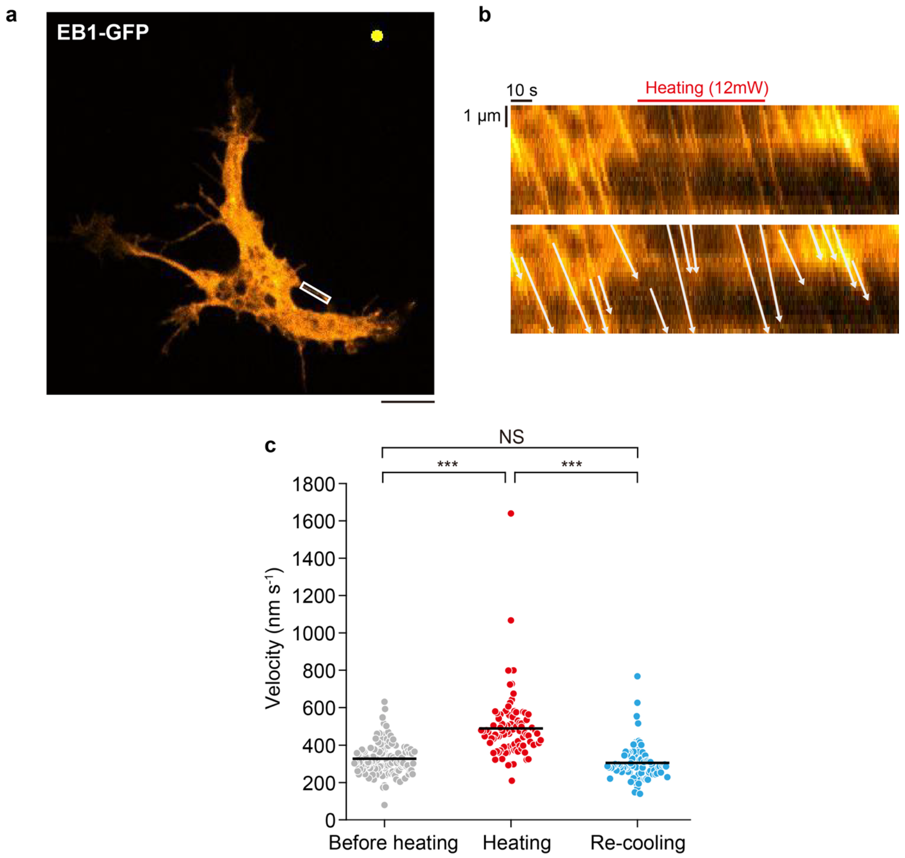

Supplementary Figure S14. Microtubule elongation in neurons during microheating. (a) Fluorescence image of EB1-GFP expression in a neuron (4 DIV). The yellow circle indicates the position of the heat source. Scale bar, 10 µm. See also Supplementary Movie S13. (b) Kymograph of EB1-GFP showing the elongation of microtubules within the area highlighted by the rectangle in (a). Top: raw image. Bottom: same image as above, but with arrows to indicate the movement of EB1-GFP. During heating (indicated by a red bar), the velocity of EB1-GFP increased, while the fluorescence intensity of EB1-GFP decreased due to thermal quenching. The T_0 was 36 °C, and the laser power was 12 mW. (c) Velocity of EB1-GFP before heating, during heating, and after re-cooling. Bars indicate average values. The velocity was compared using one-way ANOVA with Tukey-Kramer tests (***$p < 0.001$; NS, not significant).

Supplementary Table S1. Average values depicted in Fig. 2e.

T_0 (ºC)	$T_0 + \Delta T$ (ºC)	Elongation length (μm)	n
25	25	0.6 ± 1.5	58
32	32	2.5 ± 2.7	24
36	36	3.5 ± 2.7	151
25	31–33	2.3 ± 3.6	6
25	35–37	8.1 ± 4.5	4
32	35–37	2.0 ± 3.2	10
25	38–39	9.7 ± 1.8	4
36	38–39	3.6 ± 4.1	11
32	39–40	11.3 ± 1.8	4
36	39–40	7.7 ± 2.8	28

Mean ± s.d.

Supplementary Table S2. Average values depicted in Fig. 3d and in Supplementary Figs. S8 and S9.

	F_0 (n)	F_{max}/F_0		
		Unheated (n)	Heated (n)	KCl (n)
Control	336 ± 79 (44)	1.03 ± 0.02 (20)	1.35 ± 0.22 (24)	3.93 ± 1.83 (9)
30 μM ruthenium red	363 ± 136 (28)	1.00 ± 0.04 (8)	1.63 ± 0.52 (20)	-
1.8 mM EGTA	99 ± 43 (35)	1.03 ± 0.04 (17)	1.10 ± 0.08 (18)	-
30 μM EGTA-AM	270 ± 57 (33)	1.00 ± 0.01 (14)	1.16 ± 0.05 (19)	1.12 ± 0.07 (10)
50 μM BAPTA-AM	35 ± 15 (34)	1.02 ± 0.01 (15)	1.00 ± 0.04 (19)	1.24 ± 0.06 (10)

Mean ± s.d.

Supplementary Table S3. Average values depicted in Fig. 5b.

	Area change (%)			n
	Increase	Decrease	Total	
Control	15.1 ± 8.4	-12.4 ± 5.6	2.7 ± 10.6	14
10 μM cytochalasin D	6.2 ± 6.5	-10.2 ± 4.3	-4.0 ± 6.5	11
30 μg·mL^{-1} colchicine + 20 μM taxol	18.7 ± 9.4	-14.2 ± 5.3	4.5 ± 8.2	8

Mean ± s.d.

Supplementary Table S4. Average values depicted in Fig. 5c.

	$\Delta F/F_0$ of GFP-tubulin after heating	n
Control	-0.23 ± 0.10	14
10 μM cytochalasin D	-0.20 ± 0.13	9
30 μg·mL^{-1} colchicine + 20 μM taxol	0.07 ± 0.24	7

Mean ± s.d.

Supplementary Table S5. Average values depicted in Supplementary Fig.S2.

DIV	Elongation length (μm)	n
1	12.1 ± 4.4	27
2	12.0 ± 4.3	27
4	14.9 ± 5.4	28
8	16.1 ± 6.1	19

Mean ± s.d.

Supplementary Table S6. Average values depicted in Supplementary Fig. S10.

	Fluorescence intensity of rhodamine phalloidin-labelled F-actin (a.u.)	n
Control	284 ± 119	48
10 μM cytochalasin D	271 ± 56	37
10 μM latrunculin B	47 ± 20	34
0.1% DMSO	257 ± 73	38
30 μM EGTA-AM	239 ± 56	42
50 μM BAPTA-AM	211 ± 72	36

Mean ± s.d.

a.u., arbitrary units

Supplementary Table S7. Average values depicted in Supplementary Fig. S11.

	Fluorescence intensity of the Alexa Fluor 488-labelled tubulin (a.u.)	n
Control	591 ± 119	66
10 nM vinblastine	621 ± 238	43
30 $\mu g \cdot mL^{-1}$ colchicine	272 ± 86	65
10 μM nocodazole	266 ± 101	74
20 μM taxol	607 ± 134	57

Mean ± s.d.

a.u., arbitrary units

Supplementary Table S8. Average values depicted in Supplementary Fig.S13.

	Unheated (n)		Heated (n)	
	Axon	Minor neurites	Axon	Minor neurites
Acet. Tub/Tub	0.92 ± 0.10 (18)	0.59 ± 0.14 (59)	0.99 ± 0.14 (9)	0.50 ± 0.19 (9)
Tyr. Tub/Tub	0.87 ± 0.28 (31)	1.27 ± 0.37 (92)	0.80 ± 0.19 (12)	1.29 ± 0.37 (15)

Mean ± s.d.

Acet., acetylated; Tyr., tyrosinated; Tub, α-tubulin

Supplementary Methods

Cell culture

Hippocampal neurons were dissected from 18–19 day-old rat embryos (Nippon Bio-Supp. Center, Tokyo, Japan). Hippocampi cut from brains were incubated in Hank's balanced salt solution (HBSS) (Life Technologies, Thermo Fisher Scientific, Inc., Waltham, MA, USA) containing 0.02% trypsin (Life Technologies) and 100 $\mu g \cdot mL^{-1}$ DNase (Hoffmann-La Roche, Ltd., Basel, Switzerland) for 8 min at 37 °C. After adding 10% fetal bovine serum (FBS) (Life Technologies), the solution was centrifuged at 181 × *g* for 3 min at 4 °C. The supernatant was then exchanged for HBSS containing 10% FBS and 100 $\mu g \cdot mL^{-1}$ DNase. The tissue was pipetted 5 times, centrifuged at 181 × *g* for 3 min at 4 °C, and the supernatant was exchanged for dissection medium [HBSS containing 10 mM HEPES and 1 mM pyruvic acid (Sigma-Aldrich Co., St. Louis, MO), pH 7.2 (NaOH)] containing 70 $\mu g \cdot mL^{-1}$ DNase. The tissue was then pipetted 15 times and centrifuged at 181 × *g* for 3 min at 4 °C, and the supernatant was exchanged for dissection medium containing 50 $\mu g \cdot mL^{-1}$ DNase. The tissue was again pipetted 15 times and centrifuged at 181 × *g* for 5 min at 4 °C, and the supernatant was exchanged for culture medium (Neurobasal medium containing 2% B-27 supplement, 81.4 $\mu g \cdot mL^{-1}$ L-Glutamine, 100 units$\cdot mL^{-1}$ penicillin, and 100 $\mu g \cdot mL^{-1}$ streptomycin; all products were obtained from Life Technologies). The tissue was then resuspended by pipetting 20 times. The mechanically dissociated cells were passed through a cell strainer (40 μm, Becton, Dickinson and Co., Franklin Lakes, NJ, USA) and seeded on glass-bottom dishes (AGC Techno glass Co., Shizuoka, Japan). Dishes were pre-treated with collagen and poly-D-lysine using the following methods: the glass-bottom dishes were coated with 0.3 $mg \cdot mL^{-1}$ collagen (Cellmatrix Type I-C, Nitta Gelatin Inc., Osaka, Japan) diluted in 10^{-3} M HCl and incubated for 60 min at 37 °C in the presence of 5% CO_2. After removing the collagen solution and drying for 5–30 min at room temperature, the dishes were washed twice with phosphate-buffered saline [PBS (-); 137 mM NaCl, 2.7 mM KCl, 1.5 mM KH_2PO_4, 8.1 mM Na_2HPO_4] and treated with 0.1 $mg \cdot mL^{-1}$ poly-D-lysine (Merck KGaA, Darmstadt, Germany) diluted in sterilized water for 1–24 h at 37 °C in the presence of 5% CO_2. The poly-D-lysine solution was removed and the dishes were dried for 5–30 min at room temperature, washed with sterilized water three times, and filled with 2 mL of culture medium. 100 μL of culture medium containing 1.0×10^5 cells was then added to the dishes. The cells were cultured at 37 °C in the presence of 5% CO_2 for 12–24 h before observation.

Live cell imaging

For observations of Ca^{2+} dynamics, cells were incubated in culture medium containing 1 μM fluo-4-AM (Life Technologies) with or without either 30 μM EGTA-AM (Life Technologies) or 50 μM BAPTA-AM (Dojindo Laboratories, Kumamoto, Japan) for 30 min at 37 °C. The culture medium was then exchanged for HEPES-buffered saline [HBS; 140 mM NaCl, 5 mM KCl, 1 mM $MgCl_2$, 1 mM Na_2HPO_4, 10 mM HEPES, 1.8 mM $CaCl_2$, 25 mM D-glucose (pH 7.4, adjusted with NaOH)] to suppress the autofluorescence of the solution, HBS containing 30 μM ruthenium red (Sigma-Aldrich Co.), or HBS containing 1.8 mM EGTA without $CaCl_2$. The cells were then placed on the microscope 15 min prior to observation.

For observations of neurite elongation in the presence of chemical agents, the culture medium was exchanged for HBS supplemented with either 10 nM vinblastine (Sigma-Aldrich), 25 μM ciliobrevin D (Merck KGaA), 30 $\mu g \cdot mL^{-1}$ colchicine (Sigma-Aldrich), 10 μM nocodazole (Merck KGaA), 20 μM paclitaxel (taxol) (Sigma-Aldrich), 10 μM cytochalasin D (Wako Pure Chemical Industries, Ltd., Osaka, Japan), 10 μM latrunculin B (Merck KGaA), 25 μM blebbistatin (Toronto Research Chemicals Inc., Ontario, Canada), 20 μM ML-7 (Sigma-Aldrich Co.), 10 μM Y-27632 (Wako Pure Chemical Industries, Ltd.), or 30 μM ruthenium red (Sigma-Aldrich Co.). The reason exchanging the culture solution was to suppress the aggregation of chemical agents. The cells were then incubated in HBS containing inhibitor agents, or in HBS containing 1.8 mM EGTA without $CaCl_2$ for 15 min at 37 °C. For experiments using EGTA-AM and BAPTA-AM, cells were incubated in culture medium containing 0.1% DMSO, 30 μM EGTA-AM, or 50 μM BAPTA-AM for 30 min at 37 °C. The culture medium was then exchanged for HBS, and the cells were placed on the microscope 15 min prior to observation.

The inhibitor concentrations used were chosen based on the results of previous reports, indicating their appropriateness for investigating targets in rat hippocampal neurons (colchicine[1,2], nocodazole[3–5], taxol[1,3], cytochalasin D[2,5–7], latrunculin B[8], blebbistatin[5], ML-7[5,9], Y-27632[5], ruthenium red[10,11], and BAPTA-AM[12]). The concentrations of vinblastine and ciliobrevin D used were also chosen based on previous reports[13,14]. To reduce the concentration of DMSO (0.1%), the concentration of EGTA-AM used (30 μM) was lower than the typical concentration (50 μM)[12,15,16]. 1 mM fluo-4-AM, 30 $mg \cdot mL^{-1}$ colchicine, 10 mM nocodazole, 20 mM taxol, 10 mM cytochalasin D, 10 mM latrunculin B, 25 mM ciliobrevin D, 25 mM blebbistatin, 20 mM ML-7, 30 mM EGTA-AM, and 50 mM BAPTA-AM dissolved in DMSO were stored as stock solutions at -20 °C. Stocks of 10 μM vinblastine, 10 mM Y-27632, and 30 mM ruthenium red dissolved in water were also stored at -20 °C. Differences

between culture solutions (i.e., culture medium or HBS) did not affect the elongation length of the neurites during observation. The inhomogeneous patterns present in bright-field images lacking cells were considered background, and were subtracted from images of cells using ImageJ software.

The flow of extracellular solutions was visualized using 1-μm diameter FluoSpheres Carboxylate-Modified Microspheres (Life Technologies). Convection-like water flow was generated by negative pressure produced inside an open-tipped glass capillary tube placed near a cell. The negative pressure was manually controlled with a syringe pump. A similar method was utilized for application of 1 M KCl to cells by positive pressure.

Transient expression of Tubulin-GFP was performed using the CellLights Tubulin-GFP, BacMam 2.0 baculovirus expression system (Life Technologies). One day after the preparation of neurons, 20 μL of reagent was added to the 2 mL of culture medium. After 3 days, cells were treated with 0.125 $\mu g \cdot mL^{-1}$ CellMask Orange (Life Technologies) in HBS for 15 min at 37 °C to stain plasma membranes. For the observation of neurons in the presence of chemical agents, plasma membranes were stained with 0.125 $\mu g \cdot mL^{-1}$ CellMask Orange in HBS containing 20 μM taxol or 10 μM cytochalasin D for 15 min at 37 °C. The solution was then replaced with HBS containing both 30 $\mu g \cdot mL^{-1}$ colchicine and 20 μM taxol, or 10 μM cytochalasin D. The cells were then placed on a microscope for 15 min prior to observation, and the GFP and CellMask Orange were observed by confocal microscopy. EB1-GFP was expressed using Lipofectamine LTX (Life Technologies). After 3 days, the cells cultured in HBS were observed by confocal microscopy.

Immunostaining

F-actin was stained with rhodamine phalloidin (Life Technologies), as follows: cells at 1 DIV were incubated in HBS containing 0.1% DMSO, 10 μM cytochalasin D, or 10 μM latrunculin B for 30 min at 37 °C. After washing with PBS (-) containing 10 mM EGTA and 2 mM $MgCl_2$, the cells were fixed in PHEM buffer (60 mM PIPES, 25 mM HEPES, 10 mM EGTA, 2 mM $MgCl_2$) containing 4% paraformaldehyde (Wako Pure Chemical Industries), 0.25% glutaraldehyde (Wako Pure Chemical Industries), and 0.1% Triton X-100 for 15 min in a heating bucket (TAITEC Co. Saitama, Japan) at 37 °C. After washing with PBS (-), the fixed cells were incubated in PBS (-) containing 0.1 μM rhodamine-phalloidin and 0.083% bovine serum albumin (BSA; Biowest, Nuaillé, France) for 20 min in the heating bucket set at 37 °C. The cells were then washed again with PBS (-), and visualized in PBS (-).

For staining of α-tubulin, cells at 1 DIV were incubated in HBS containing 10

nM vinblastine, 30 μg·mL^{-1} colchicine, 10 μM nocodazole, and 20 μM taxol for 30 min at 37 °C. The fixation method used was the same as that used for staining of F-actin. After washing with PBS (-), the fixed cells were incubated in blocking solution [PBS (-) containing 1.5% BSA] for 60 min at room temperature. After blocking, the cells were incubated in blocking solution containing rabbit anti-α-tubulin antibody (1:300, ab15246, Abcam plc) for 60 min at room temperature. After washing with blocking solution, the cells were incubated in blocking solution containing Alexa Fluor 488-conjugated goat anti-rabbit IgG (1:300, A-11008, Life Technologies) for 60 min at room temperature. Cells were then washed with PBS (-), and visualized in PBS (-).

To investigate the effects of microheating on modification of tubulins, acetylated, tyrosinated, and total α-tubulin were stained. Cells at 3 DIV in culture medium were locally heated (laser power; 11 mW) at 36 °C. Within 30 min after heating, the cells were fixed and blocked, as described above. After blocking for 60 min, the cells were incubated in blocking solution containing rabbit anti-α-tubulin antibody (1:1000, ab15246, Abcam plc) and either mouse anti-acetylated α-tubulin antibody (1:1000, ab24610, Abcam plc) or rat anti-tyrosinated α-tubulin antibody (YL1/2) (1:1000, ab6160, Abcam plc) for 60 min at room temperature. After washing with blocking solution, the cells were incubated in blocking solution containing both Alexa Fluor 594-conjugated goat anti-rabbit IgG (1:1000, A-11012, Life Technologies) and Alexa Fluor 488-conjugated goat anti-mouse IgG (1:1000, A-11001, Life Technologies), or both Alexa Fluor 488-conjugated goat anti-rabbit IgG (1:1000, A-11008, Life Technologies) and Alexa Fluor 555-conjugated goat anti-rat IgG (1:1000, A-21434, Life Technologies) for 60 min at room temperature. After washing with PBS (-), the cells were visualized in PBS (-).

Supplementary References

1. Aoyagi, A., Saito, H. & Abe, K. Differential effects of microtubule inhibitors on axonal branching and elongation of cultured rat hippocampal neurons. *Jpn. J. Pharmacol.* **68,** 223–6 (1995).

2. Shan, J., Munro, T. P., Barbarese, E., Carson, J. H. & Smith, R. A molecular mechanism for mRNA trafficking in neuronal dendrites. *J. Neurosci.* **23,** 8859–66 (2003).

3. Witte, H., Neukirchen, D. & Bradke, F. Microtubule stabilization specifies initial neuronal polarization. *J. Cell Biol.* **180,** 619–32 (2008).

4. Kollins, K. M., Hu, J., Bridgman, P. C., Huang, Y. Q. & Gallo, G. Myosin-II negatively regulates minor process extension and the temporal development of neuronal polarity. *Dev. Neurobiol.* **69,** 279–98 (2009).

5. Roland, A. B. *et al.* Cannabinoid-induced actomyosin contractility shapes neuronal morphology and growth. *eLife* **3,** e03159 (2014).

6. Bradke, F. The role of local actin instability in axon formation. *Science* **283,** 1931–4 (1999).

7. Allison, D. W., Gelfand, V. I., Spector, I. & Craig, A. M. Role of actin in anchoring postsynaptic receptors in cultured hippocampal neurons: differential attachment of NMDA versus AMPA receptors. *J. Neurosci.* **18,** 2423–36 (1998).

8. Korkotian, E. & Segal, M. Spike-associated fast contraction of dendritic spines in cultured hippocampal neurons. *Neuron* **30,** 751–8 (2001).

9. Tokuoka, H. & Goda, Y. Myosin light chain kinase is not a regulator of synaptic vesicle trafficking during repetitive exocytosis in cultured hippocampal neurons. *J. Neurosci.* **26,** 11606–14 (2006).

10. Trudeau, L. E., Emery, D. G. & Haydon, P. G. Direct modulation of the secretory machinery underlies PKA-dependent synaptic facilitation in hippocampal neurons. *Neuron* **17,** 789–97 (1996).

11. Crawford, D. C., Moulder, K. L., Gereau, R. W., Story, G. M. & Mennerick, S. Comparative effects of heterologous TRPV1 and TRPM8 expression in rat hippocampal neurons. *PLoS One* **4,** e8166 (2009).

12. Abramov, A. Y. & Duchen, M. R. Mechanisms underlying the loss of mitochondrial membrane potential in glutamate excitotoxicity. *Biochim. Biophys. Acta* **1777,** 953–64 (2008).

13. Lu, W., Fox, P., Lakonishok, M., Davidson, M. W. & Gelfand, V. I. Initial neurite outgrowth in drosophila neurons is driven by kinesin-powered microtubule sliding. *Curr. Biol.* **23,** 1018–23 (2013).

14. Lu, W., Lakonishok, M. & Gelfand, V. I. Kinesin-1-powered microtubule sliding initiates axonal regeneration in Drosophila cultured neurons. *Mol. Biol. Cell* **26,** 1296–307 (2015).

15. Xie, M. *et al.* Facilitation versus depression in cultured hippocampal neurons determined by targeting of Ca^{2+} channel $Ca_v\beta_4$ versus $Ca_v\beta_2$ subunits to synaptic terminals. *J. Cell Biol.* **178,** 489–502 (2007).

16. Larsson, M. *et al.* Functional and anatomical identification of a vesicular transporter mediating neuronal ATP release. *Cereb. Cortex* **22,** 1203–14 (2012).

Supplementary Movie captions

Supplementary Movie S1. Neurite outgrowth induced by microheating.
The cell membrane was stained with CellMask, and cells were observed by confocal microscopy. The yellow circle indicates the position of the heat source. The T_0 was 36 °C, and the laser power was 12 mW. Scale bar, 10 μm. See also Fig. 1d.

Supplementary Movie S2. Neurite outgrowth and branching induced by microheating.
The yellow circle indicates the position of the heat source. The T_0 was 36 °C, and the laser power was 11 mW. Scale bar, 10 μm. Inhomogeneous patterns present in images lacking cells were subtracted. See also Supplementary Fig. S1a.

Supplementary Movie S3. Morphological changes in growth cones induced by microheating.
The morphology of the growth cone changed dramatically during heating. The yellow circle indicates the position of the heat source. The T_0 was 36 °C, and the laser power was 11 mW. Scale bar, 10 μm. Inhomogeneous patterns present in images lacking cells were subtracted. See also Supplementary Fig. S1b.

Supplementary Movie S4. Neurite regrowth triggered by microheating after injury.
A glass capillary tube was utilized to remove a part of a neurite. The injured neurite formed a small growth cone and resumed to grow within 30 min after injury. Microheating for 60 s induced remarkable regrowth of the neurite. The open circle indicates the position of the heat source. The T_0 was 36 °C, and the laser power was 11 mW. Scale bar, 10 μm. See also Supplementary Fig. S3.

Supplementary Movie S5. Formation of physical connections during microheating.
The yellow circle indicates the position of the heat source. The T_0 was 36 °C, and the laser power was 11 mW. Scale bar, 20 μm. Inhomogeneous patterns present in images lacking cells were subtracted. See also Supplementary Fig. S4.

Supplementary Movie S6. Neurite outgrowth in the presence of a high temperature gradient.
Yellow circles indicate the isotherm (ΔT = 4, 5, 6, 7, 8, and 9 °C). The T_0 was 36 °C, and the laser power was 18 mW. Scale bar, 20 μm. Inhomogeneous patterns present in images lacking cells were subtracted. See also Fig. 2a.

Supplementary Movie S7. Neurite outgrowth in the presence of a low temperature gradient.
Yellow circles indicate the isotherm (ΔT = 2, 3, and 4 °C). The T_0 was 36 °C, and the laser power was 9 mW. Scale bar, 20 μm. Inhomogeneous patterns present in images lacking cells were subtracted. See also Fig. 2b.

Supplementary Movie S8. Neurite outgrowth is not triggered by convection flow.
Left: small particles are fluorescent beads. First, the medium was aspirated using a glass pipette, and water flow was induced (0–60 s). The amount of neurite outgrowth observed was unremarkable. Right: microheating (0–60 s) of the same cell resulted in reduced water flow, but rapid neurite outgrowth. The yellow circle indicates the position of the heat source. The T_0 was 36 °C, and the laser power and 11 mW. Scale bar, 10 μm. See also Supplementary Fig. S6.

Supplementary Movie S9. Ca^{2+} dynamics during microheating.
Intracellular Ca^{2+} was visualized using the fluorescent Ca^{2+} indicator fluo-4. The infrared laser was turned on at 0 s and turned off at 60 s. The bright spot at the lower left is the position of heat source. The T_0 was 36 °C, and the laser power was 11 mW. Scale bar, 10 μm. See also Fig. 3a.

Supplementary Movie S10. Microtubule dynamics during microheating.
Confocal images of microtubules in cells expressing tubulin-GFP (green) and treated with the cell membrane stain CellMask (magenta). The yellow circle indicates the position of the heat source. The T_0 was 36 °C, and the laser power was 22 mW. Scale bar, 10 μm. See also Fig. 5a.

Supplementary Movie S11. Microtubule dynamics during microheating in the presence of 10 μM cytochalasin D.
Confocal images of microtubules in cells expressing tubulin-GFP (green) and treated with the cell membrane stain CellMask (magenta). The yellow circle indicates the position of the heat source. The T_0 was 36 °C, and the laser power was 22 mW. Scale bar, 10 μm. See also Supplementary Fig. S12a.

Supplementary Movie S12. Microtubule dynamics during microheating in the presence of 30 $\mu g \cdot mL^{-1}$ colchicine and 20 μM taxol.
Confocal images of microtubules in cells expressing tubulin-GFP (green) and treated

with the cell membrane stain CellMask (magenta). The yellow circle indicates the position of the heat source. The T_0 was 36 °C, and the laser power was 22 mW. Scale bar, 10 μm. See also Supplementary Fig. S12b.

Supplementary Movie S13. Microtubule elongation in neurons during microheating.

Confocal image of a neuron (4 DIV) expressing EB1-GFP. The yellow circle indicates the position of the heat source. The T_0 was 36 °C, and the laser power was 12 mW. Scale bar, 10 μm. See also Supplementary Fig. S14.
